# Understanding the regulation of overwintering diapause molecular mechanisms in *Culex pipiens pallens* through comparative proteomics

**DOI:** 10.1038/s41598-019-42961-w

**Published:** 2019-04-24

**Authors:** Chongxing Zhang, Dongdong Wei, Guihong Shi, Xiaoli Huang, Peng Cheng, Gongzhen Liu, Xiuxia Guo, Lijuan Liu, Huaiwei Wang, Feng Miao, Maoqing Gong

**Affiliations:** 1 Shandong Institute of Parasitic Diseases, Shandong First Medical University & Shandong Academy of Medical Sciences, Jining, Shandong 272033 P.R. China; 2 Collaborative Innovation Center for the Origin and Control of Emerging Infectious Diseases, Taishan Medical University, Taian, Shandong 271000 P.R. China; 3 Shanghai MHelix BioTech Co., Ltd., 271000 Shanghai, P.R. China

**Keywords:** Protein-protein interaction networks, Entomology

## Abstract

To reveal overwintering dormancy (diapause) mechanisms of *Culex pipiens pallens* (L.), global protein expression differences at three separate time points represent nondiapause, diapause preparation and overwintering diapause phases of *Cx*. *pipiens pallens* were compared using iTRAQ. *Cx*. *pipiens pallens* females accumulate more lipid droplets during diapause preparation and overwintering diapause maintenance than during the nondiapause phase. A total of 1030 proteins were identified, among which 1020 were quantified and compared. Gene Ontology, Kyoto Encyclopedia of Genes and Genomes (KEGG), Domain and Clusters of Orthologous Groups (COG) analyses revealed key groups of proteins, pathways and domains differentially regulated during diapause preparation and overwintering diapause maintenance phases in this mosquito, including major shifts in energy production and conversion, fatty acid metabolism, the citrate (TCA) cycle, and the cytoskeletal reorganization pathway. Our results provide novel insight into the molecular bases of diapause in mosquitoes and corroborate previously reported diapause-associated features in invertebrates. More interestingly, the phototransduction pathway exists in *Cx*. *pipiens pallens*, in particular, actin, rather than other proteins, appears to have substantial role in diapause regulation. In addition, the differential changes in calmodulin protein expression in each stage implicate its important regulatory role of the *Cx*. *pipiens pallens* biological clock. Finally, 24 proteins were selected for verification of differential expression using a parallel reaction monitoring strategy. The findings of this study provide a unique opportunity to explore the molecular modifications underlying diapause in mosquitoes and might therefore enable the future design and development of novel genetic tools for improving management strategies in mosquitoes.

## Introduction

*Culex pipiens pallens* is the primary vector of *Wuchereria bancrofti filariasis*, *Japanese encephalitis* virus, West Nile virus and *Rickettsia felis* in China^1,2^. *Cx*. *pipiens pallens* mosquitoes overwinter in a state of adulthood diapause^3^, and overwintering mosquitoes play significant roles in the population dynamics of these species and in the spread and prevalence of mosquito-borne diseases^4^. Therefore, clarifying overwintering diapause mechanisms is essential for improving mosquito management strategies.

The results of previous studies have increased our knowledge of the molecular regulatory mechanisms of diapause preparation, maintenance and termination, revealing several physiological themes that appear to be shared across the diapause response of multiple insect species, including shifts in metabolism^5–8^, increased lipid synthesis and storage^9–11^, upregulation of stress-response genes^12,13^, hormonal regulation during diapause induction^12,14–20^, and changes in insulin signalling^8,19,21–23^. Most previous studies on the molecular mechanisms of diapause regulation have been performed at the transcriptional level, leading to exciting progress with regard to the transcriptional basis of diapause in several insect taxa^7,21,24–35^. However, an overall relatively low correlation between the expression levels of mRNAs and the abundance of various proteins has been reported in a comparison of prediapause and non-diapause locust eggs^31^. As diapause has evolved independently multiple times during the evolutionary history of insects, it is unclear to what extent metabolic patterns are conserved across species that exhibit the same or different diapause syndromes. Overall, a single suite of mechanisms regulating diapause across all species and developmental stages is unlikely^36^, yet our understanding of the molecular mechanisms involved in generating, maintaining, and breaking diapause is still highly fragmentary^14^. This is especially true because proteomic information on diapause is limited and the mechanism by which organisms measure and interpret photoperiod remains completely unresolved and controversial. Studies on diapause-related changes in expression of proteins relevant to physiological processes can facilitate a better understanding of the specific mechanisms involved in diapause-driven physiological changes and their physiological responses to physical environmental cues as well as elusive seasonal adaption.

With the advent of highly advanced proteomic platforms based on isobaric tags for relative and absolute quantification (iTRAQ)^37^, proteomics allows for screening differentially expressed proteins at a large scale, which ultimately provides a direct measurement of protein expression levels and insight into the activity of relevant proteins. Changes in protein expression during diapause have mainly been explored using 2-dimensional gel electrophoresis (2-DE)^38–43^ and shotgun proteomics^44^ and using Itraq^31,45^ on other insects. In contrast, there are no reports of proteomic analysis of diapause in mosquitoes thus far.

In this study, iTRAQ was used to investigate the proteome of *Cx*. *pipiens pallens* collected in July 2016 (representing nondiapausing, SUM), November 2016 (diapause preparation, BW) and March 15–19, 2017 (the overwinter diapause maintenance stage of female mosquitoes, AW). The results reveal novel insight into the metabolic processes occurring in these phases and enhance our understanding of the mechanisms underlying overwintering diapause in mosquitoes. These findings may serve as a foundation and provide a platform for developing novel vector control methods based on genetic or chemical disruption of such key traits^46^.

## Results

### Ovarian development, lipid storage status, protein identification and expression of different protein (EDP) screening

**Figure 1:**
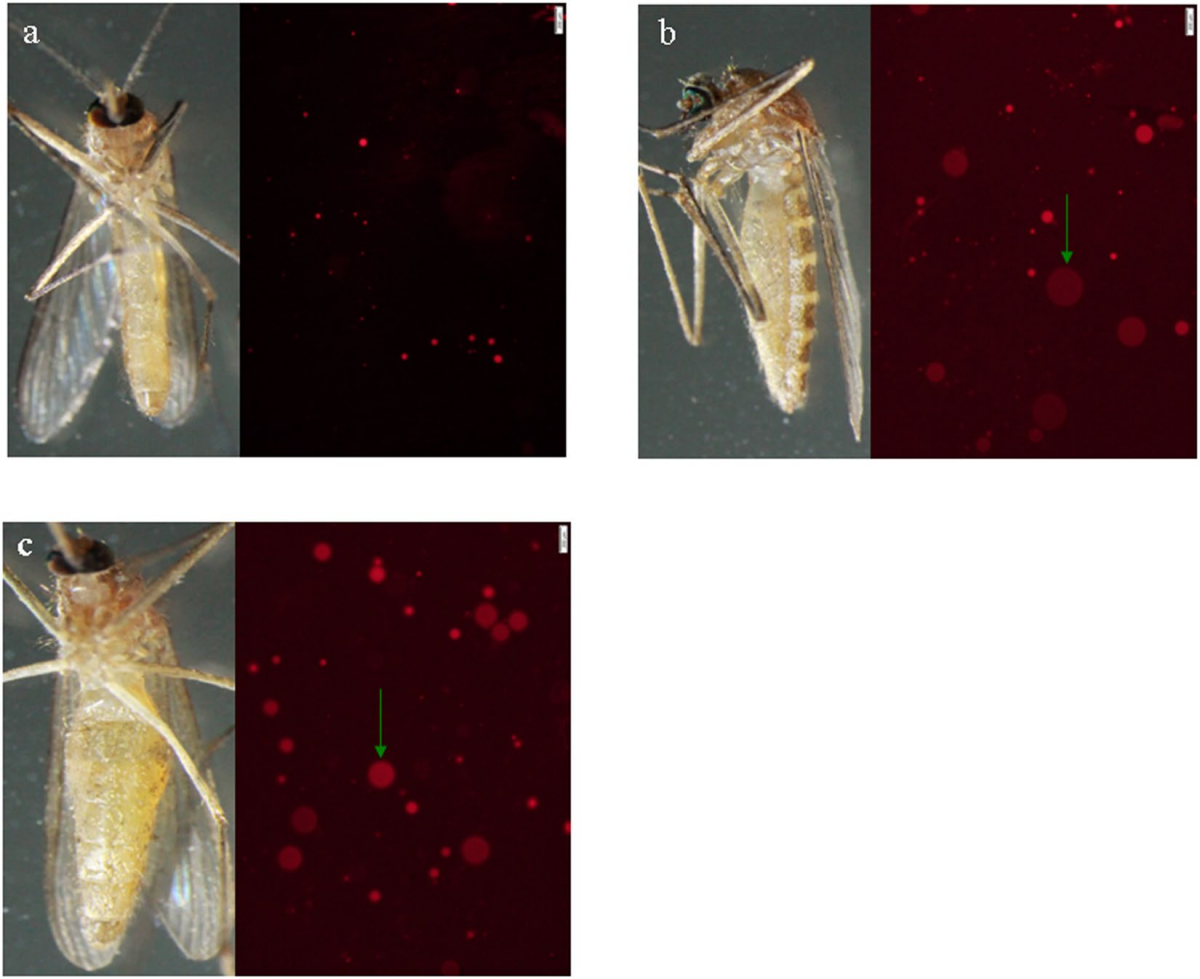
(**a**) Slim females and Nile Red staining of lipid droplets in SUM adult females. (**b**,**c**) Fat females and Nile Red staining of lipid droplets in BW and AW adult females, respectively. Green arrow: larger lipid droplets.

**Figure 2:**
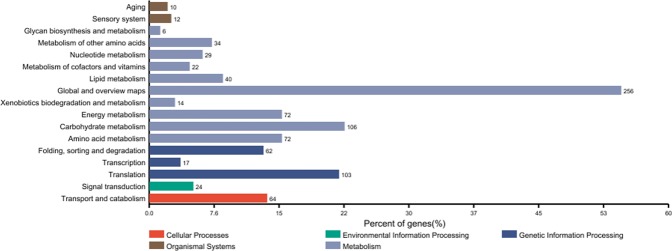
Statistics of differential proteins for KEGG annotation. The x-axis shows the percentage of proteins contained in different metabolic types; the y-axis shows the different metabolic types and number of proteins involved.

Ovarian development and lipid storage status were monitored (Table 1, Fig. 1). A total of 1,030 proteins were identified, among which quantitative information was obtained for 1,020. All the proteins were annotated by Gene Ontology, KEGG, COG, and Domain and Subcellular Location analyses (Supplementary Table 1). Interestingly, ageing (10 members) and sensory system (12 members) pathways were identified in KEGG pathway analysis (Fig. 2). There were 244 and 174 differentially expressed proteins identified between AW vs. BW and BW vs. SUM, respectively.

**Table 1:** Dissection of *Cx*. *pipiens pallens*.

Date		No. of dissections	Percent of fat bodies (%)			Percent of ovarian development (%)				
			+	++	+++	I	II	III	IV	V
2016	Nov. 1–8	105	8.6	43.8	47.6	98.1	1.9	0	0	0
	Nov. 12–20	88	5.7	30.7	63.7	97.7	0	0	2.3	0
	Nov. 23–29	48	0	29.1	70.9	100	0	0	0	0
	Dec. 3–7	79	0	11.4	88.6	100	0	0	0	0
	Dec. 11–20	76	1.3	9.2	89.5	100	0	0	0	0
	Dec. 25–30	135	0	10.4	89.6	100	0	0	0	0
2017	Jan. 4–9	120	0	5.8	94.2	100	0	0	0	0
	Jan. 11–19	146	0	5.5	94.5	100	0	0	0	0
	Jan. 21–22	86	0	1.2	98.8	100	0	0	0	0
	Feb. 8	5	0	0	100	100	0	0	0	0
	Mar. 15–19	12	0	33.3	66.7	100	0	0	0	0
	Mar. 23–28	12	41.7	50	8.3	0	100	0	0	0
	Apr. 1–9	20	60	40	0	0	95	0	0	5
	Apr. 11–16	6	100	0	0	16.7	0	0	0	83.3
	Total	938	4.1	16.1	79.8	95.6	3.5	0	0.2	0.7

“+”: A few lipid droplets can be seen under the microscope.

“+++”: More lipid droplets under the microscope.

“++++”: A stretch of lipid droplets under the microscope.

### Protein expression patterns and function Clustering

**Figure 3:**
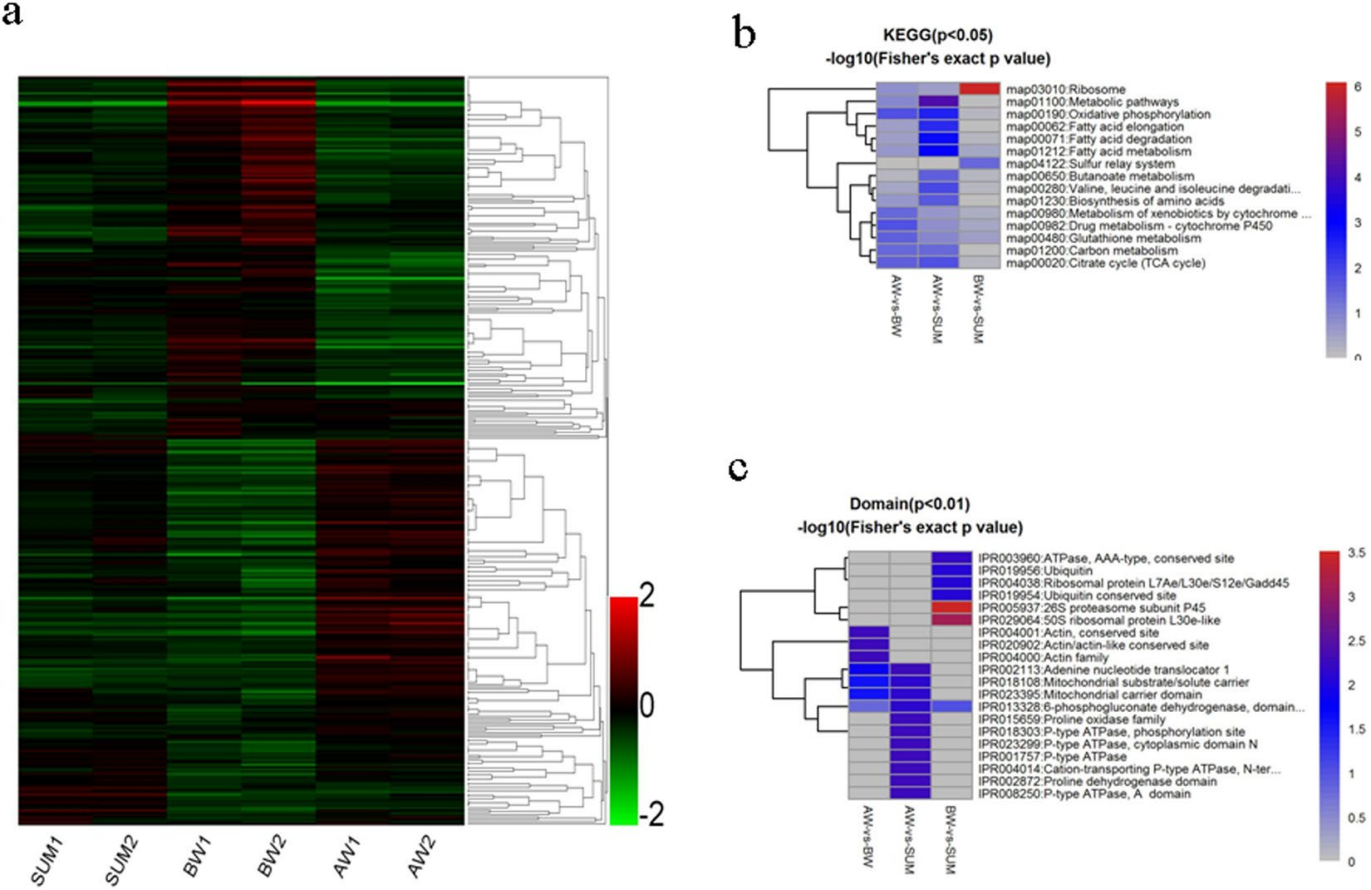
Clustering of protein expression patterns and functional enrichment. (**a**) Differential protein relative expression matrix heatmap of the three groups; the colour in the figure indicates the relative expression level of the protein in the sample. The red colour indicates that the protein has a higher expression level in the sample, and the green colour represents a lower expression level. Colour bars represent specific expression abundance. (**b,c**) Functional enrichment-based clustering for protein groups in KEGG and Domain. Clustering of the results of differential protein enrichment in different groups. The colour −log10 (Fisher exact test p value) represents the credibility of enrichment.

**Figure 4:**
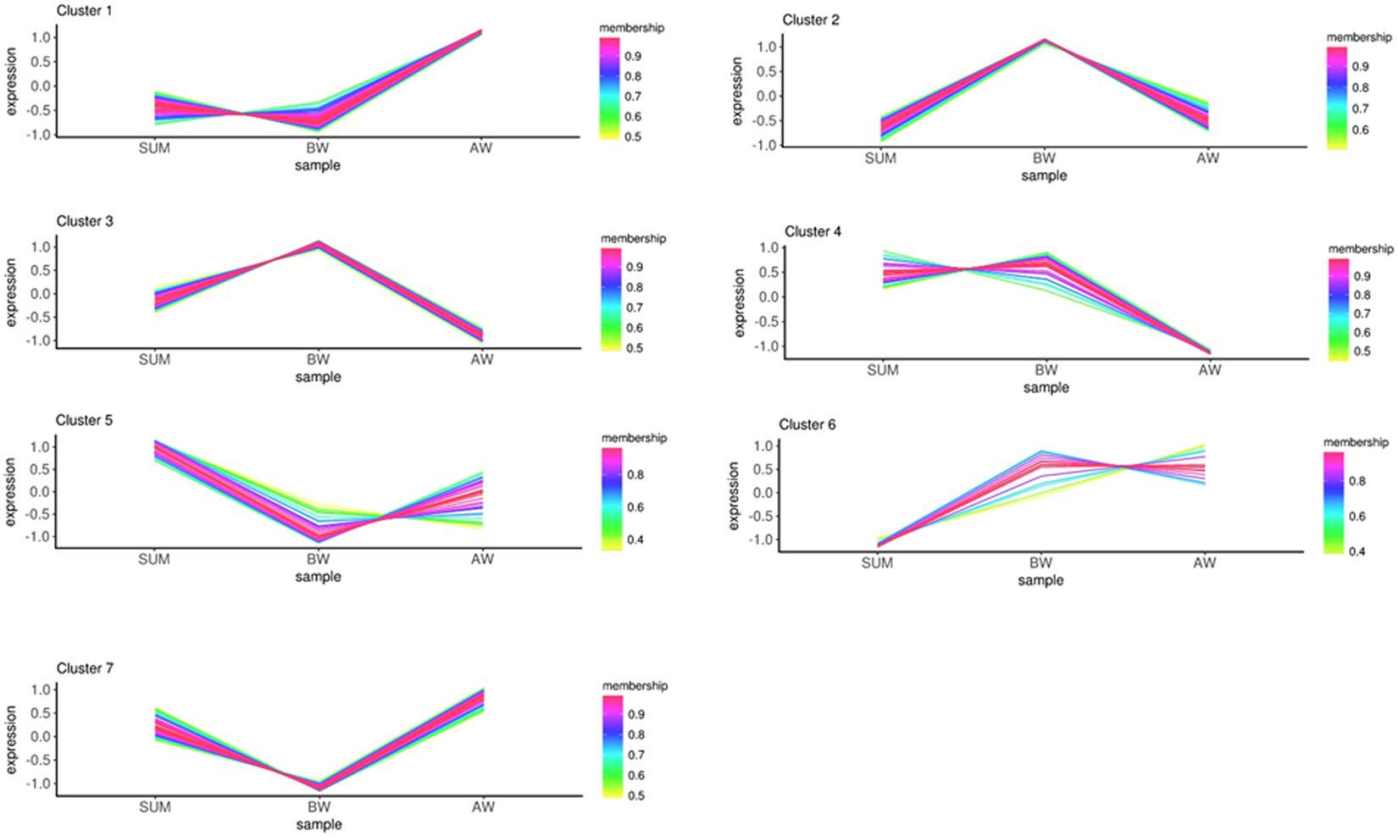
Comparison of protein expression pattern clusters between SUM, BW and AW. For the expression trend line graph of each sub-cluster, the x-axis is the comparison sample group, and the y-axis is the relative expression level of the protein in the group of samples. Each line in the figure represents a protein, and the different colour representations show the relationship between relative expression and the mean value. Each graph shows one type of expression pattern, a trend that reflects changes in the expression of this group of proteins.

**Figure 5:**
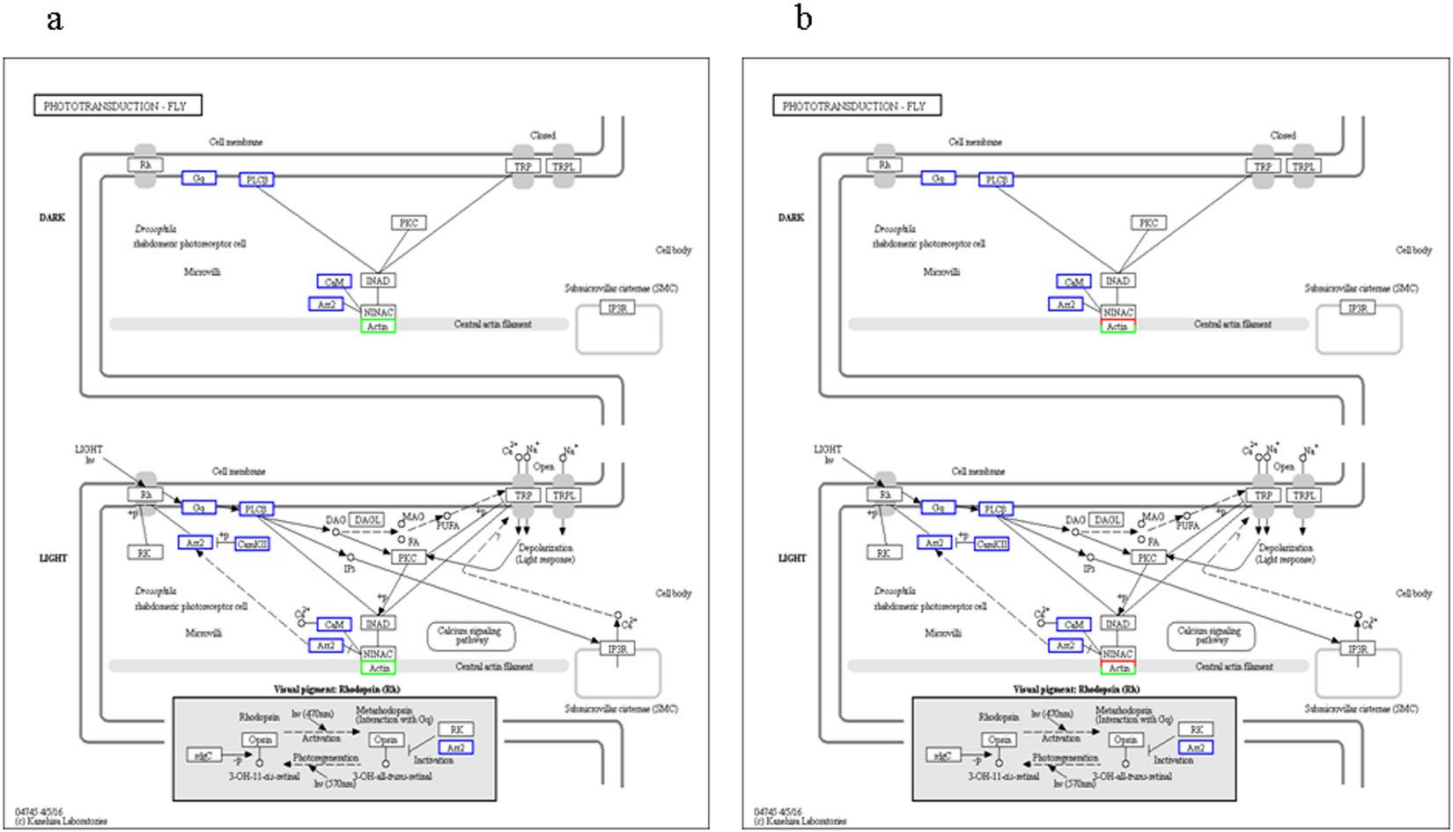
(**a**) Representative KEGG phototransduction pathway in BW vs. SUM, map04745, and (**b**) phototransduction pathway in AW vs. BW, map04745; the rectangular nodes in the figure represent gene products. The blue border belongs to the background proteins, and the white colour indicates proteins not identified in this experiment. The red/green colours in the figure indicate to the differentially expressed proteins detected in this study, with red representing upregulated proteins and green downregulated proteins. Half red and half green indicates both upregulated and downregulated proteins for that gene product^74–76^.

We employed hierarchical clustering to characterize changes in expression during the diapause preparation and overwintering diapause maintenance phases compared to a nondiapause phase control. All quantified proteins revealed clearly distinguishable increasing or decreasing expression during BW and AW (Fig. 3). Comparing the pattern of expression of different proteins between SUM, BW and AW, almost all of the identified proteins overlapped between stages, presenting a continuous dynamic of quantitative changes at different time points. Although the fold enrichment of each protein was quite small, almost all protein enrichment changes at the peak and the lowest valley were in BW, indicating that the abundance of various proteins changes in response to preparation for overwintering in *Cx*. *pipiens pallens* and that the diapause preparation process alters the normal developmental pathway into an alternative one. The individual ecological phenomenon of overwintering diapause was found to be very important in the long-term evolution of mosquitoes (Fig. 4). Interestingly, the phototransduction pathway exists, implicating an important regulatory role in this mosquito (Fig. 5). Furthermore, the differential changes in calmodulin protein expression in each group suggests a critical regulatory role associated with *Cx*. *pipiens pallens’s* biological clock. Of note is the differentially expressed protein actin in the phototransduction pathway. Actin-87E (B0WEY5), actin (B0FBP2) and actin (A0A173GY68) were downregulated in BW vs. SUM but upregulated in AW vs. BW; actin-2 (B0WEY6) was downregulated in AW vs. BW. These findings indicate that actin may play an important role in the regulation of this process in the phototransduction pathway.

### PPI network analysis

**Figure 6:**
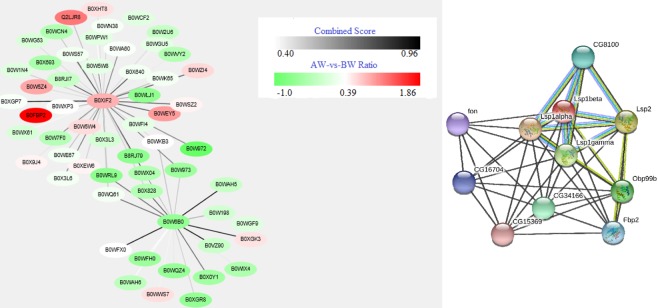
Calmodulin (B0XIF2), glutathione s-transferase (B0W6B0) and LSP-1 beta (B0XHC4) were revealed by Cytoscape. (**a**) Each circle in the figure represents a protein; the ratio value is the log2 (FC) value of the comparison group AW vs. BW, and the shade of the colour between the genes represents the combined score. (**b**) The interactions between expressed proteins are indicated by the connecting line, with the thickness of an edge indicating the confidence in that associations (thicker line indicates more evidence for interaction). Different line colours represent the types of evidence for the association: green = neighbourhood; red = gene fusion; pink = experiments; light green = text mining; blue = cooccurrence; dark blue = coexpression; purple = homology. Circle nodes indicate different proteins.

We then used STRING, which reveals functional associations between two groups, to further analyse the functional correlation of differentially expressed proteins between each group (Fig. 6). The mean combined score was 0.589; the maximum combined score between calmodulin (B0XIF2) and serine/threonine-protein phosphatase (B0XGP7) was 0.931, and the maximum combined score between glutathione S-transferase 1–6 (B0W6B0) and glutathione S-transferase (B0VZ90) was 0.952. The maximum and minimum ratio values for the AW and BW groups were 1.85 (CPIJ006534-PA, B0FBP2 (actin)) and −1.0 (CPIJ003588-PA, B0W972 (ubiquitin)), respectively.

### Verification and validation of differentially expressed proteins by a parallel reaction monitoring strategy

**Figure 7:**
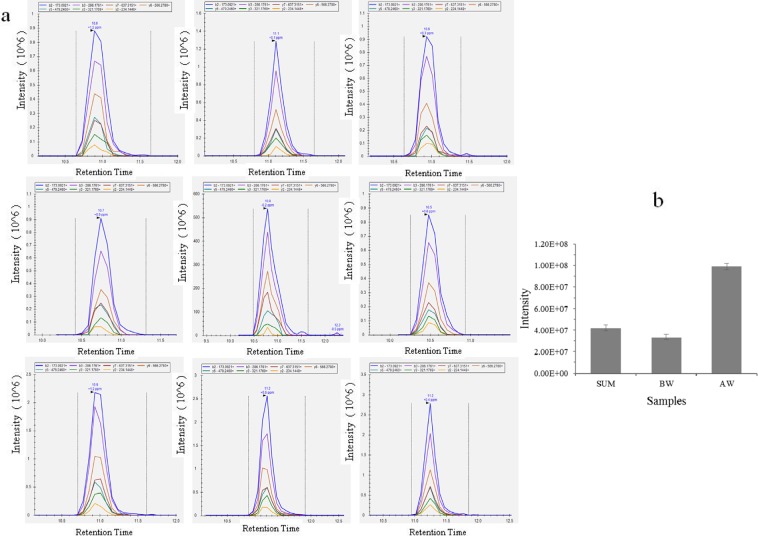
(**a**) PRM for the abundance of glycerol-3-phosphate dehydrogenase was determined. Graphs displaying chromatograms of fragment ions extracted from the peptide TALVEADDFASGTSSK. The mass measurement error and retention time of the most intense transition are annotated above the peak. (**b**) Quantification performance of PRM analyses of the glycerol-3-phosphate dehydrogenase peptide. Highly consistent results were obtained, reflecting the excellent agreement between the intensities of peptides.

Using a parallel reaction monitoring (PRM)-quantifiable strategy, 24 differentially expressed proteins of *Cx*. *pipiens pallens* were confirmed. All proteins, peptides, and measured peptide peak areas are listed in Supplementary Table 2 and Fig. 7.

## Discussion

Because most insects do not feed during the long period of diapause and increase lipid stores are often associated with hardiness against metabolic stress^8,47–50^, fat storage in diapause preparation and regulation of its utilization are important for successful overwintering. From November 2016, most *Cx*. *pipiens pallens* female mosquitoes entered a diapause preparation phase, accumulated more lipid droplets, arrested follicular development at stage I and maintained this stage until March 15–19, 2017. AW and BW female *Cx*. *pipiens pallens* mosquitoes were conspicuously larger and fatter than their SUM counterparts, and larger lipid droplets were observed in AW and BW. Indeed, no large fat lipid droplets were found in the SUM groups, revealing a dramatic increase in fat storage and the number of fat body cells in AW and BW of *Cx*. *pipiens pallens* (Table 1, Fig. 1), consistent with previous findings that *Culex pipiens* pupae and adults are larger and contain more fat than do their nondiapausing counterparts programmed for adult diapause^51^.

Because diapause is often accompanied by a decrease in metabolism, in accordance with reduced metabolism and energy production and an overall metabolic reduction, KEGG pathway enrichment analysis in BW vs. SUM specifically revealed downregulation of metabolic pathways, fatty acid biosynthesis, oxidative phosphorylation, starch and sucrose metabolism and phagosome pathways (Supplementary Figs 1–5). As reported previously in *Cx*. *pipiens*, fatty acid synthase is upregulated and fat metabolism genes, including multiple kinetic classes and genes involved in *β*-oxidation, an energy-generation step, are suppressed in early diapause^21^. Such findings are consistent with global suppression of the expression of proteins involved in fatty acid synthesis found in recent high-throughput genomic studies during other forms of hypometabolism, such as hibernation in ground squirrels^52^ and the mosquito *Aedes albopictus*^27^.

Redirection of energy production is among the defining characteristics of diapause^53,54^; however, a previous study associated upregulation of genes involved in fatty acid synthesis and sequestration at the molecular level in *Cx*. *pipiens* at the onset of diapause^21^. How can these two views be reconciled? It should be noted that the above study examined only a single time point during diapause; in contrast, the more comprehensive investigation of the functional roles of proteins in our study, along with our evaluation of the full course of diapause, would likely result in a clearer understanding of the intriguing diapause phenotype. Overall, this pattern suggests that overall metabolic and energy production rates are lower in BW, thus enabling the mosquito to economically utilize energy.

Energy metabolism is crucial for the survival of diapausing insects throughout winter. Consistent with these considerations, energy demands are fuelled by lipid stores, and *Cx*. *pipiens pallens* females begin to utilize triacylglyceride stores, convert stored fat to free fatty acids, and transport these fatty acids to mitochondria to meet energetic needs. We found that 43 proteins associated with energy production and conversion were upregulated during prolonged overwintering in female mosquitoes, indicating a general activation of lipid consumption. As shown in Supplementary Fig. 6, the six most enriched GO terms regarding biological processes of upregulated proteins are related to the oxidation-reduction process, generation of precursor metabolites and energy, energy derivation by oxidation of organic compounds, the single-organism metabolic process, the single-organism process, and cellular respiration, with high fold enrichment (log2) and a significant Fisher’s exact test p-value (-log10). KEGG pathway enrichment analysis provided additional evidence, specifically regarding the upregulation of metabolic pathways, oxidative phosphorylation, the citrate cycle (TCA cycle), carbon metabolism, fatty acid metabolism, starch and sucrose metabolism, fatty acid biosynthesis, glycolysis/gluconeogenesis and phototransduction (Supplementary Figs 7–15). In particular, differential gene expression analysis in diapausing *Cx*. *pipien* females provided evidence for increases in two separately upregulated glycogen-debranching enzymes involved in glycolysis and gluconeogenesis metabolism^30^. Moreover, examination of the enriched proteins in the over-represented energy production category and oxidative phosphorylation pathway showed changes at the protein level in overwintering diapause conditions and increased levels of several nicotinamide adenine dinucleotide dehydrogenase (NADH) subunits (complex), suggesting that overwintering diapause enhances energy production through the oxidative phosphorylation pathway.

In AW vs. BW, the components of the TCA pathway (Supplementary Fig. 10) upregulated in overwintering diapause are largely proteins that are involved directly in oxidative metabolism in the mitochondria and in alternative mitochondrial or cytosolic reactions that support glycolysis and gluconeogenesis. Conversely, the proteins acting in the initial steps in the TCA cycle involving the conversion of pyruvate from glycolysis to acetyl-CoA appear to be suppressed (pyruvate dehydrogenase E1 component subunit alpha, B0W2T1). This step is a key gateway for entry of carbon flux from the glycolytic pathway into the mitochondria and is responsible for catalysing the conversion of pyruvate and acyl-CoA to form acetyl-CoA. Isocitrate dehydrogenase [NADP] (B0VZM6), which was observed to be downregulated in AW vs. BW, is a key enzyme regulating the reaction between isocitric acid and NAD, a rate-limiting step of the TCA cycle. Downregulation of pyruvate dehydrogenase E1 component subunit alpha and isocitrate dehydrogenase [NADP] in overwintering diapause would enable mosquitoes to economically utilize energy reserves.

Our experiments also indicate that expression of certain cytoskeletal proteins is affected by overwintering diapause in *Cx*. *pipiens pallens*, as a relatively large number of cytoskeleton-associated proteins with a diversity of functions showed highly differential abundance in AW vs. BW and BW vs. SUM (Fig. 3c). For example, actin-87E (B0WEY5), actin (A0A173GY68, B0FBP2), myosin light chain 1 (B0X1Z6), tropomodulin (B0XBH8) and fimbrin/plastin (B0WA60) and tubulin beta chain (Q15G69) were downregulated in BW vs. SUM. Examination of component-enriched lists in the cytoskeleton category in AW and BW showed upregulation of spectrin alpha chain (B0X640), actin-87E (B0WEY5), tropomyosin invertebrate (B0X3L6), actin (A0A173GY68, B0FBP2), myosin light chain 1 (B0X1Z6), talin-1 (B0WXE5), and tubulin alpha chain (B0WK65). Actins constitute a large family of proteins with specific localization and regulation, and thus, levels of different actins might be elevated while levels of others might be decreased concomitantly during diapause^55^. These results correspond to observations in diapausing adult *Cx*. *pipiens*, whereby actin was found to be upregulated in early diapause but returned to low levels by late diapause^56^. According to data for wheat (*Triticum aestivum*), actin downregulation may contribute to the increased cold tolerance associated with dormancy^57^. In addition, a brain-specific actin was reported to be downregulated during pharate larval diapause of the gypsy moth *Lymantria dispar*^58^. Interestingly, in *Drosophila americana*, actinD1 expression levels have also been shown to reflect the observed differences in the life span of both diapausing and non-diapausing flies^59^. In our study, actin, conserved site, actin family, actin/actin-like conserved site were the most enriched domains for upregulated proteins in AW vs. BW (Supplementary Fig. 16). Taken together, our observations confirm the notion that cytoskeleton proteins show altered expression and cellular cytoskeletal rearrangement, suggesting fortification of structural components in response to overwinter diapausing and leading to increased cold tolerance and desiccation resistance in diapausing *Cx*. *pipiens pallens*.

Proline, a well-known energy source and cryoprotectant in some insects^6^, increases cold tolerance in insects; in fact upregulation of proline is commonly considered a potentially important source of metabolic fuel for overwintering^60–62^. In this study, pyrroline-5-carboxylate dehydrogenase (B0WKF4) and proline oxidase (B0WRS5) were upregulated in AW vs. BW, and upregulation of pyrroline-5-carboxylate reductase has also been reported for *Culex pipiens*^30^.

Calmodulin, which can bind the second messenger calcium and affect a number of target proteins, is an important regulatory protein that adjusts a wide range of cellular processes and development^63^. Chilling from 25 °C to 0 °C evoked a 40% increase in intracellular calcium concentrations in the tracheal cells of the freeze-tolerant *Eurosta solidaginis* and increased the activity of calcium/calmodulin-dependent protein kinase II (CaMKII)^64^. Additionally, calmodulin levels were decreased in pupal diapause induction in *Helicoverpa armigera*^65^, and calmodulin and calmodulin protein kinase II were decreased in larval diapause initiation^40^. Calcineurin A and FK506-binding protein, which are related to Ca^2+^ release, were uniquely identified in diapausing *Bombyx mori* eggs^66^. Moreover, the calcium ATPase gene was upregulated in 3-day-diapausing *Tetranychus urticae*^67^.

Interestingly, in the present study, the phototransduction signalling network that perceives day length and ultimately translates seasonal information into the developmental programme we recognize as diapause in *Cx*. *pipiens pallens* exhibited differential changes in calmodulin protein (B0XIF2) expression (Fig. 5) in each group, implicating a regulatory role for calmodulin in phototransduction pathways. Actin is a critical link in the regulation of proteins in the phototransduction pathway response to seasonal alteration in *Cx*. *pipiens pallens*, though details and insight into how calmodulin and actin affect diapause at both local and systemic levels and to what extent calmodulin signals are involved in diapauses of different developmental stages are lacking.

Diapause appears to have evolved multiple times in insect lineages, and photoperiodic signals are effective at evoking diapause only during the sensitive period of an insect’s life cycle, which is species specific and may occur well before the actual diapause stage^14^. Thus, we can anticipate species variation in how this calmodulin signalling pathway is exploited for regulating diapause in different species. The exciting prospect is that this is the first report of an association between calmodulin and actin with the mosquito biological clock, with connections to insect diapause at many levels, and these findings provide a first step towards understanding the protein regulatory network of phototransduction in diapausing mosquitoes. Thus, our results provide a unique opportunity to explore the molecular modifications underlying overwintering diapause in *Cx*. *pipiens pallens*, a major goal for deciphering the molecular mechanisms of diapause.

Additional interesting differences include larval serum proteins (Fig. 6), which are storage proteins that participate in metamorphosis by supplying energy and serving as a source of amino acids. Furthermore, protein LSP-1 beta (B0XHC4) was dramatically increased in BW, with a considerably lower abundance of protein LSP-1 beta in AW, in which its expression level was even lower than in SUM. Therefore, *Cx*. *pipiens pallens* likely synthesizes many serum haemolymph proteins not only to accelerate fat storage but also to strengthen its immune system sensitivity in the high-efficiency ingestion phage in preparation for overwinter. Although protein LSP-1 beta has been indicated as among the primary serum proteins in different physiological phages *of Cx*. *pipiens pallens*, including the larva, its function remains unknown. In addition, our results support the conclusion that increased expression of the LSP-1 beta protein in BW suggest that these proteins may serve as new targets for continued research towards understanding diapause in *Cx*. *pipiens pallens* and for developing genetic tools for improving mosquito management strategies.

Glutathione s-transferase (B0W6B0) (Supplementary Fig. 17), a stress response protein, was found to be highly expressed in the spruce budworm *Choristoneura fumiferana* throughout diapause, though it levels declined at the end of diapause^68^. Glutathione s-transferase is also involved in xenobiotic biodegradation and metabolism, and *Cx*. *pipiens pallens* virulence varies among SUM, BW and AW.

## Conclusions

In summary, this study represents the first large-scale investigation of global protein expression differences during the diapause preparation and the overwintering diapause maintenance phases of the mosquito *Cx*. *pipiens pallens*. Using iTRAQ, we found major metabolic changes in several pathways and expression changes in important proteins involved in overwintering diapause regulation, revealing new insight into the physiology and regulation of overwintering diapause in *Cx*. *pipiens pallens*. These results provide comprehensive proteome insight and expand our understanding of the molecular mechanisms influencing overwintering diapause in *Cx*. *pipiens pallens*. This is the first study to identify the involvement of calmodulin, actin, and the phototransduction pathway throughout overwintering diapause in *Cx*. *pipiens pallens*, and LSP-1 beta, glutathione s-transferase may also have roles in this process. We anticipate that these results will prove useful in probing the molecular events that may be common to diapause in insects of different taxa and developmental stages.

## Materials and Methods

### Mosquito collection, dissection, and fat body staining with Nile red

Summer non-diapausing female *Cx*. *pipiens pallens* mosquitoes were collected in Weifang District in July 2016. *Cx*. *pipiens pallens* mosquitoes in diapause preparation and overwintering diapause maintenance stages were also collected from November 2016 until the 16^th^ of April 2017 in Weifang District, Shandong Province until most females had terminated diapause. Collections were conducted during daylight hours from various resting sites, including animal sheds, barns, drains, large resting boxes, and other locations in and around people’s houses^3^; the samples were brought to the insectary after morphological identification. For each sampling occasion, some *Cx*. *Pipiens pallens* females were dissected under a dissecting microscope to determine their diapause status, and lipid storage status was monitored according to Zhao’s study^69^. The fat bodies were smashed using an anatomical needle and divided into 4 types: disperse, conglobation, small block, and large block. The number of lipid droplets was observed under a microscope with a 400× field of vision. Lipid droplets <50, 50–100, 100–200 and 200–400 were classified as minute, less, medium and more, respectively. Ovarian developmental stages were defined according to methods described by Spielman and Wong^70^ and Sim and Denlinger^19^. Fat content was monitored by Nile Red staining^19^. Both dissected and un-dissected mosquito samples were sorted into pools, frozen in liquid nitrogen and preserved for further analysis at −80 °C.

### Protein extraction

For protein extraction, *Cx*. *pipiens pallens* mosquitoes in the diapause preparation (collected in November 2016) and overwintering diapause maintenance (collected in February 2017) stages (each from 4~5 adult females) were added to 1:50 (W/V) lysis buffer (8 M urea, 2 mM EDTA, 10 mM DTT and 1% protease inhibitor cocktail) and homogenized thoroughly with a tissue grinder. The samples were sonicated for 3 min and centrifuged at 13,000 × g (4 °C) for 10 min to remove debris, and the protein in the supernatant was precipitated with cold acetone for 3 h at −20 °C. After centrifugation at 4 °C and 12,000 × g for 10 min, the protein pellets was redissolved with urea buffer (8 M urea, 100 mM TEAB). The protein concentration was determined using a Modified Bradford Protein Assay Kit according to the manufacturer’s instructions.

### Protein reduction, alkylation, isobaric labelling and sample cleanup

For digestion, 100 μg protein from each sample was first reduced with 10 mM DTT at 37 °C for 60 min and then alkylated with 25 mM iodoacetamide (IAM) at room temperature for 45 min in darkness. The protein pool of each sample was digested with Sequencing Grade Modified Trypsin with a ratio of protein:trypsin of 50:1 at 37 °C overnight and 100:1 for a second digestion for 4 h. After trypsin digestion, peptides were desalted using a Strata X SPE column, vacuum-dried, reconstituted in 25 μL 500 mM TEAB and processed according to the manufacturer’s protocol for the 8-plex iTRAQ kit (Applied Biosystems, Inc.)^71^. Briefly, one unit of iTRAQ reagent was added to the peptide solution after thawing and dissolving in 50 μL isopropanol. The peptide mixtures were incubated for 2 h at room temperature and then pooled and dried by vacuum centrifugation. The dried and labelled peptides were reconstituted with HPLC solution A (2% ACN, pH 10) and then fractionated by high-pH reverse-phase HPLC using a Waters Bridge Peptide BEH C18 (130 Å, 3.5 μm, 4.6 × 250 mm). Briefly, peptides were first separated by a gradient of 2% to 98% acetonitrile in pH 10 at a speed of 0.5 mL/min over 88 min into 60 fractions. The peptides were then combined into 20 fractions and dried by vacuum centrifugation. The peptide fractions were desalted using a Ziptip C18 according to the manufacturer’s instructions. The samples were dried under vacuum and stored at −20 °C until MS analyses were performed.

### High-resolution LC-MS/MS analysis

NanoLC 1000 LC-MS/MS analysis was performed using a Proxeon EASY-nLC 1000 coupled to an LTQ-Orbitrap Elite. Trypsin digestion fractions were reconstituted in 0.1% FA and directly loaded onto a reversed-phase pre-column (Acclaim PepMap^®^100C18, 3 μm, 100 Å, 75 μm × 2 cm) at 5 μL/min in 100% solvent A (0.1 M acetic acid in water). Next, peptides eluted from the trap column were loaded onto a reversed-phase analytical column (Acclaim PepMap^®^ RSLC C18, 2 μm, 100 Å, 50 μm × 15 cm). The gradient comprised an increase from 10% to 35% solvent B (0.1% FA in 98% ACN) over 60 min, 35% to 50% in 10 min and climbing to 100% in 5 min at a constant flow rate of 250 nL/min on an EASY-nLC 1000 system.

The eluent was sprayed via an NSI source at an electrospray voltage of 1.8 kV and then analysed by tandem mass spectrometry (MS/MS) using a LTQ-Orbitrap Elite. The mass spectrometer was operated in data-dependent mode, automatically switching between MS and MS/MS. Full-scan MS spectra (from m/z 350 to 1800) were acquired in the Orbitrap with a resolution of 60,000. Ion fragments were detected in the Orbitrap at a resolution of 15,000, and the 20 most intense precursors were selected for subsequent decision tree-based ion trap HCD fragmentation at a collision energy of 38% above a threshold ion count of 300 in the MS survey scan with 30.0-s dynamic exclusion. Full width at half maximum (FHMW) at 400 m/z using an AGC setting of 1e6 ions was used, and the fixed first mass was set as 100 m/z.

### Parallel reaction monitoring analysis

A label-free targeted parallel reaction monitoring (PRM) method^72^ was adopted to verify the reliability of our label-based proteomics. A total of 24 differentially expressed proteins, including one internal standard housekeeping protein, were chosen. For the SUM, BW, and AW groups, equal amounts of protein samples were analysed for semi-quantitative measurements, and each group underwent three replications. Peak areas were extracted from PRM mass spectrum data using Skyline software^73^.

### Data analysis

The resulting MS/MS raw data were searched against the *Culex* Taxonomy database (Taxon identifier: 7174, include 21082 protein sequences) downloaded from Uniprot using Sequest software integration in Proteome Discoverer (version 1.3, Thermo Scientific). Trypsin was chosen as the enzyme, and two missed cleavages were allowed. Carbamidomethylation (C) was set as a fixed modification, and oxidation (M) and acetylation in N-Term were set as variable modifications. The searches were performed using a peptide mass tolerance of 20 ppm and a product ion tolerance of 0.02 Da, resulting in a 1% false discovery rate (FDR).

The Gene Ontology (GO) annotation proteome was derived from the UniProt-GOA database (www.http://ebi.ac.uk/GOA/). After obtaining the ID of a protein based on a protein search, the ID was converted into the ID of the UniProtKB database; GO annotation information for *Culex quinquefasciatus* was located based on the UniProKB ID (ftp://ftp.pir.georgetown.edu/databases/idmapping/uniprot_idmapping/idmapping_selected.tab.gz). Among the 1030 proteins identified, 841 were annotated. KEGG Pathway is part of the Kyoto Encyclopedia of Genes and Genomes (KEGG) database^74–76^, which is a reference database for pathway mapping. KEGG Pathway analyses of identified proteins were extracted using the Search pathway tool in the KEGG Mapper platform (http://www.genome.jp/kegg/mapper.html); 711 proteins had corresponding KO numbers in the KEGG database with the species *Culex quinquefasciatus*, and 510 KO numbers were mapped to pathways. Proteins were identified by BLAST alignment and the COG protein database to predict function and conduct statistical analyses with a p value < 1e^−5^. Protein domain annotation was assessed using InterProScan^77^. GO, KEGG pathway and Domain enrichment of differential protein expression for all identified proteins was evaluated using the two-tailed Fisher’s exact test. Correction for multiple hypothesis testing was carried out under standard FDR control methods, and pathways with a corrected p value < 0.05 were considered the most significant. A heatmap was generated by the software Heml 1.0.3^78^. The expression level of the differentially expressed proteins was taken as log2 for relative expression; the historical clustering method had an average linkage, and the similarity metric was the Pearson distance. The expression pattern of the protein was completed using the timeclust function in the R package ‘TCseq’. We also present the STRING network of some known/novel protein-protein interactions as an evidence view by using the String 9.0 (Search Tool for the Retrieval of In-teracting Genes/Proteins) database of physical and functional interactions (http://string-db.org/)^79^. Among 244 proteins, 199 were found in the STRING database for the species *Culex quinquefasciatus*. We selected the combined score >0.400 between nodes for PPI with Cytoscape (version 3.0), part of which is shown in the paper but only for important proteins.

## Supplementary information

Supplement Information

Supplementary Table 1

Supplementary Table 2
