# Supplementary material for: Understanding the regulation of overwintering diapause molecular mechanisms in Culex pipiens pallens through comparative proteomics
Source: Sci Rep. 2019 Apr 24;9:6485. doi: 10.1038/s41598-019-42961-w (PMC6482188; doi:10.1038/s41598-019-42961-w)
Supplement: Supplementary file 1 — Supplement Information [file 41598_2019_42961_MOESM1_ESM.pdf]

Supplementary information:

Title

Understanding the regulation of overwintering diapause molecular mechanisms in *Culex pipiens pallens* through comparative proteomics

Author List

Chongxing Zhang chongxingzhang@aliyun.com

Dongdong Wei 78172792@qq.com

Guihong Shi ghshi@yahoo.com

Xiaoli Huang huangxl@mhelix.cn

Peng Cheng cp1978@aliyun.com

Xiuxia Guo guoguoxiua@163.com

Gongzhen Liu gongzhenliu@126.com

Lijuan Liu jj8liu@sina.com

Huaiwei Wang whw2138@126.com

Feng Miao mfcdc@163.com

Maoqing Gong gmq1965@aliyun.com

1 Shandong Institute of Parasitic Diseases, Shandong Academy of Medical Sciences, Jining, Shandong 272033, P. R. China

2 Collaborative Innovation Center for the Origin and Control of Emerging Infectious Diseases, Taishan Medical University, Taian, Shandong 271000, P. R. China

3 Shanghai MHelix BioTech Co., Ltd.

Figure Legends

Supplementary Table 1. Proteins identified in female adult *Cx. pipiens pallens* using iTRAQ.

Supplementary Table 2. PRM verification analysis of overwintering diapause-related proteins.

Supplementary figure 1. Protein overview of complete metabolism in biological systems in BW vs. SUM, map01100[75-77]
Green indicates metabolic pathways in which proteins are downregulated; red indicates metabolic pathways in which proteins are upregulated; blue indicates metabolic pathways in which proteins were quantified but do not participate in up/down regulation.

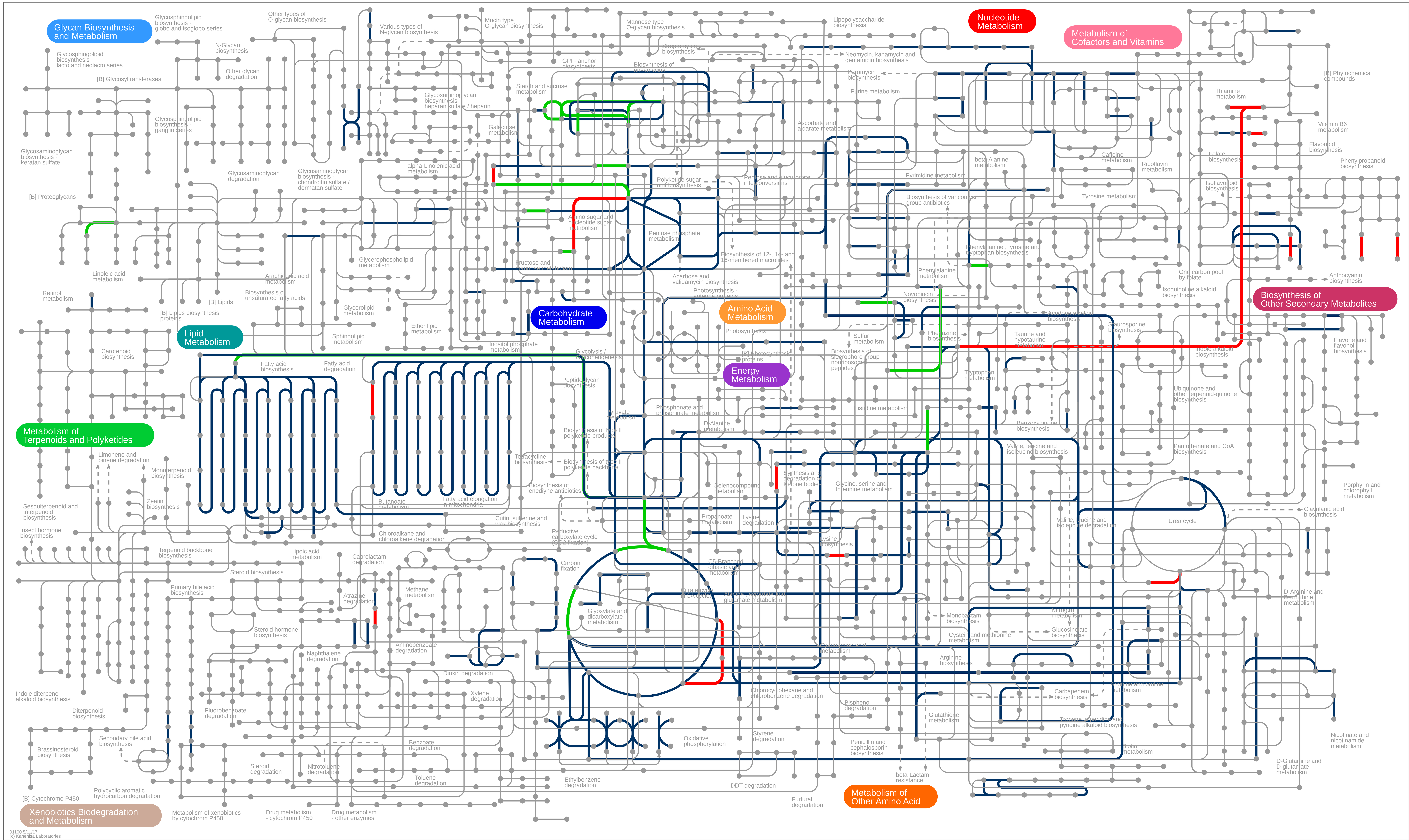

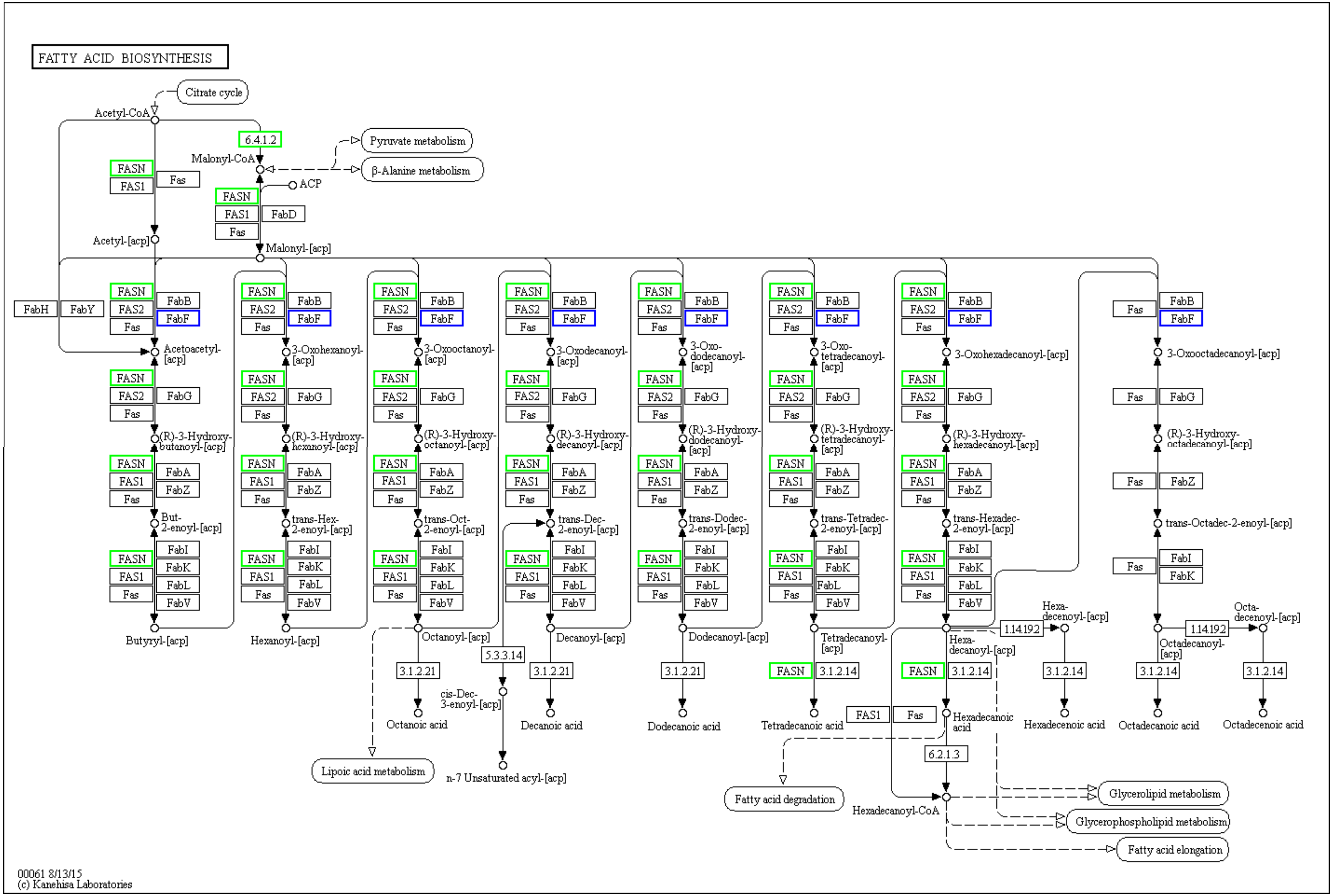

Supplementary figure 2. Annotated KEGG map for fatty acid biosynthesis in BW vs. SUM, map00061[75-77]

The blue border belongs to the background protein, and the black border indicates the protein was not identified in this experiment. The red/green colour in the figure marks the differentially expressed proteins detected in this experiment, in which red represents upregulated proteins and green represents downregulated proteins. Half red and half green indicates the protein is both upregulated and downregulated (the same meaning as in this manuscript).

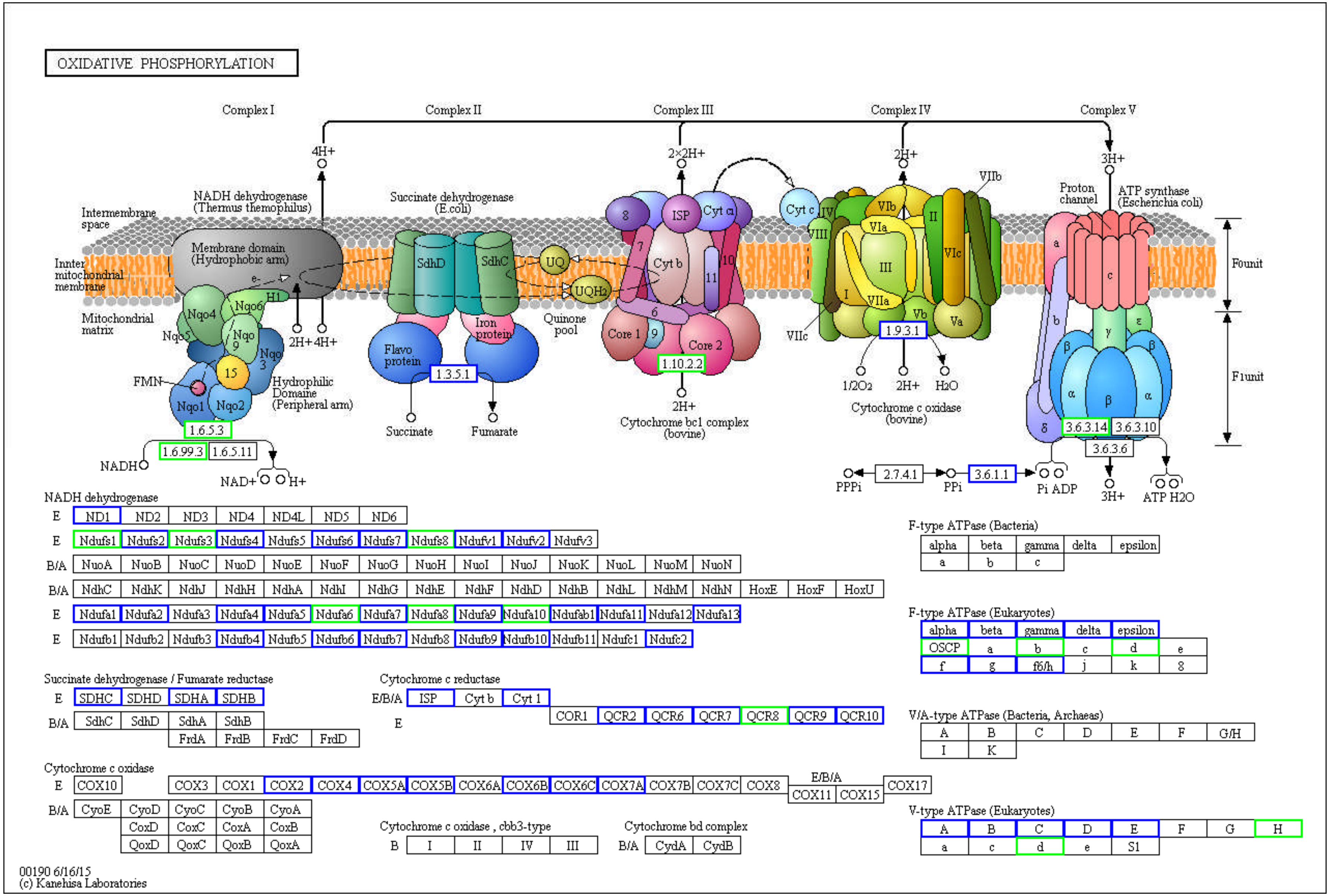

Supplementary figure 3. Annotated KEGG map for oxidative phosphorylation in BW vs. SUM, map00190[75-77]

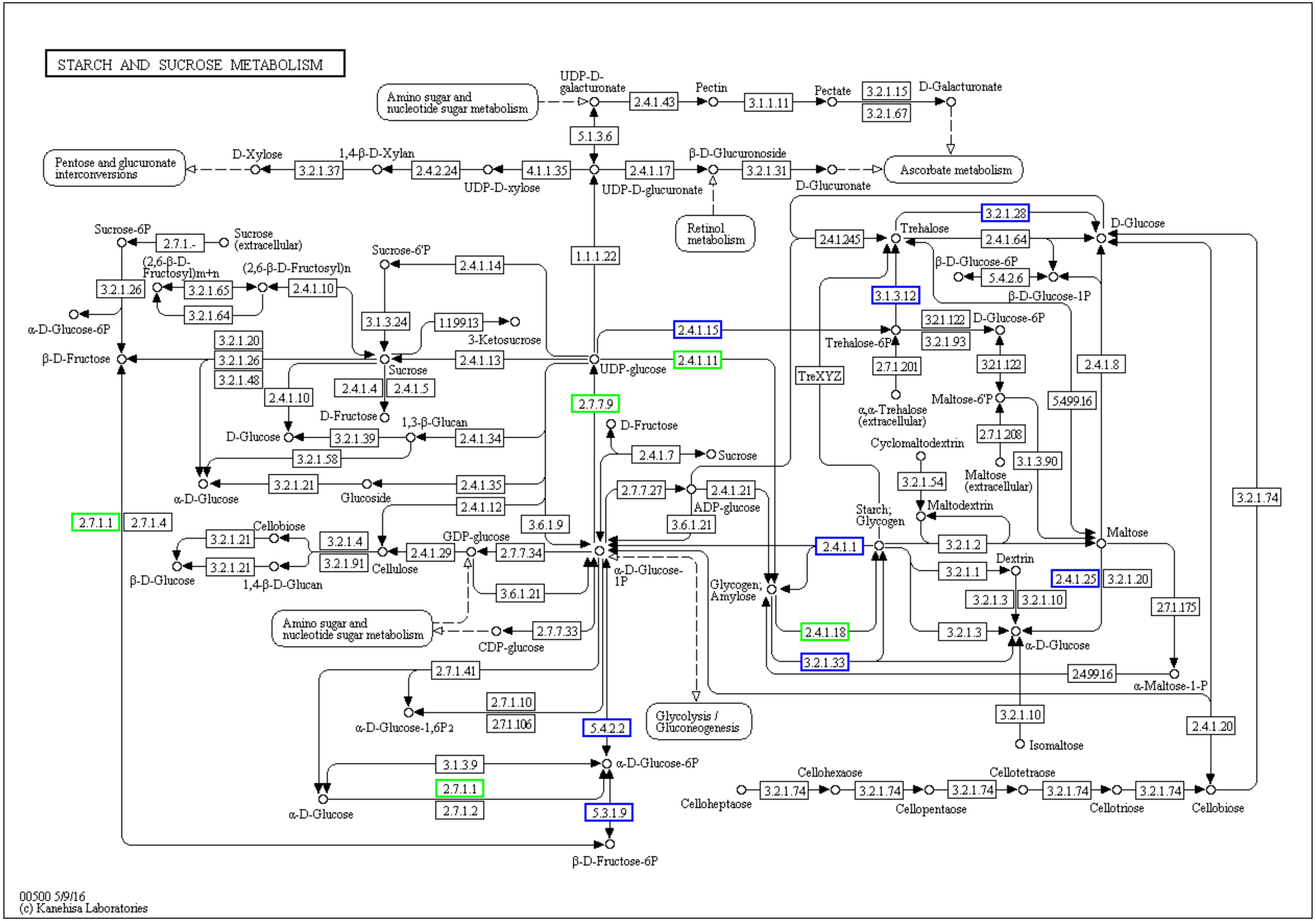

Supplementary figure 4. Annotated KEGG map for starch and sucrose metabolism in BW vs. SUM, map00500[75-77]

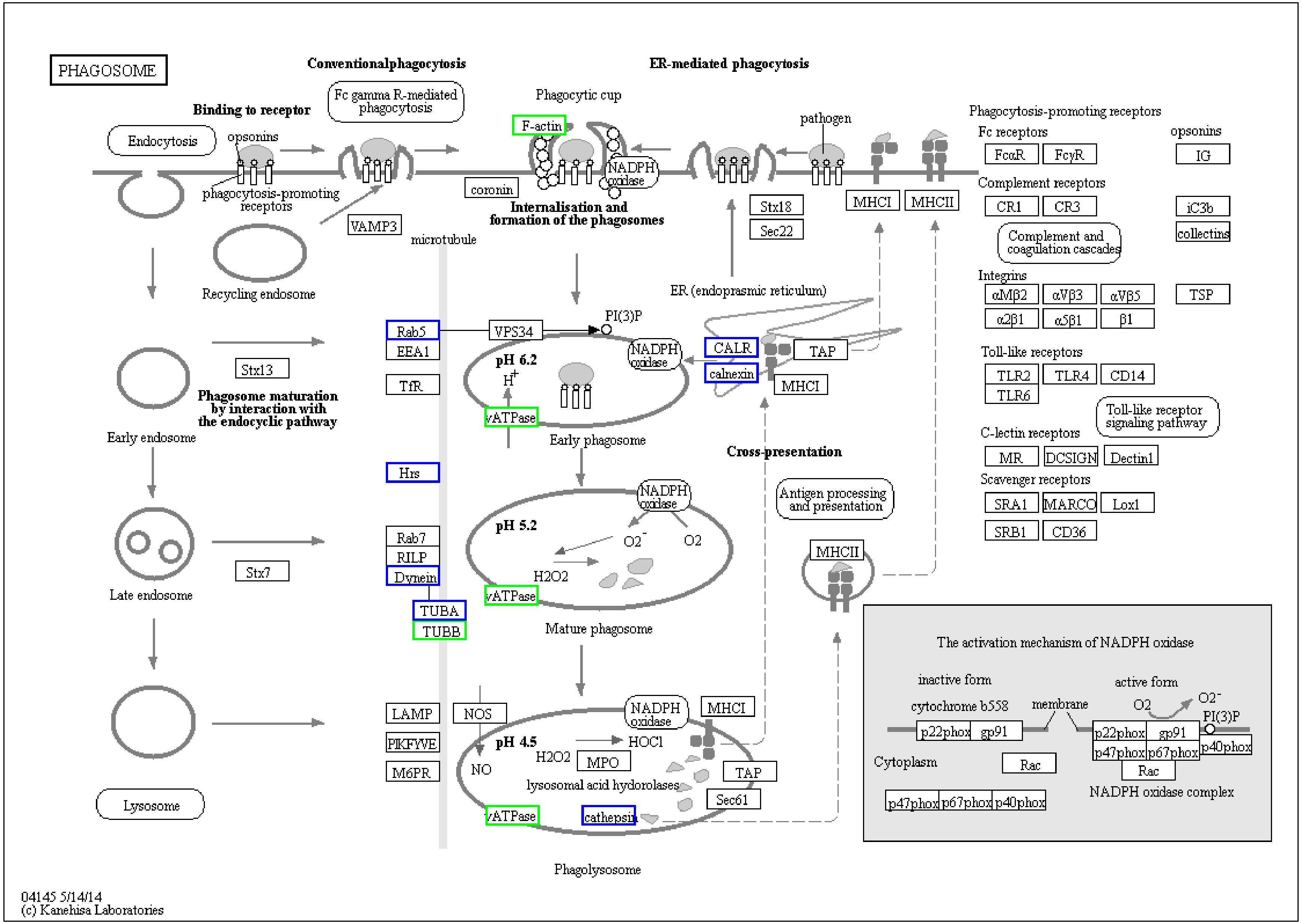

Supplementary figure 5. Annotated KEGG map for phagosomes in BW vs. SUM, map04145[75-77]

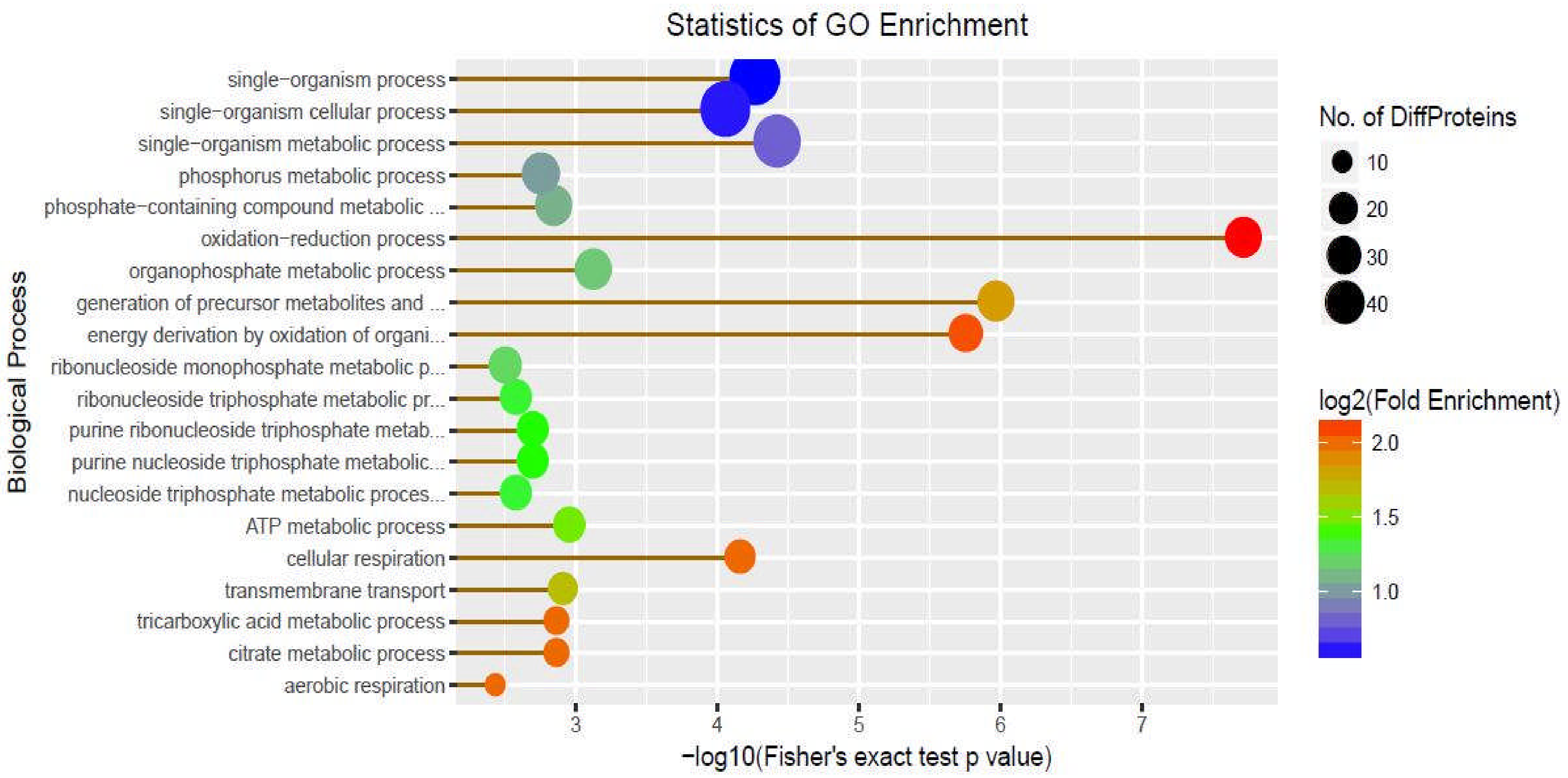

Supplementary figure 6. Visualization of significantly enriched GO terms in AW vs. BW

Upregulated proteins in the biological process category according to Fisher's extract test. The Number of Diffproteins is the number of differentially expressed proteins enriched in GO terms; the GO enrichment fold is shown on the log2 scale in a colour gradient.

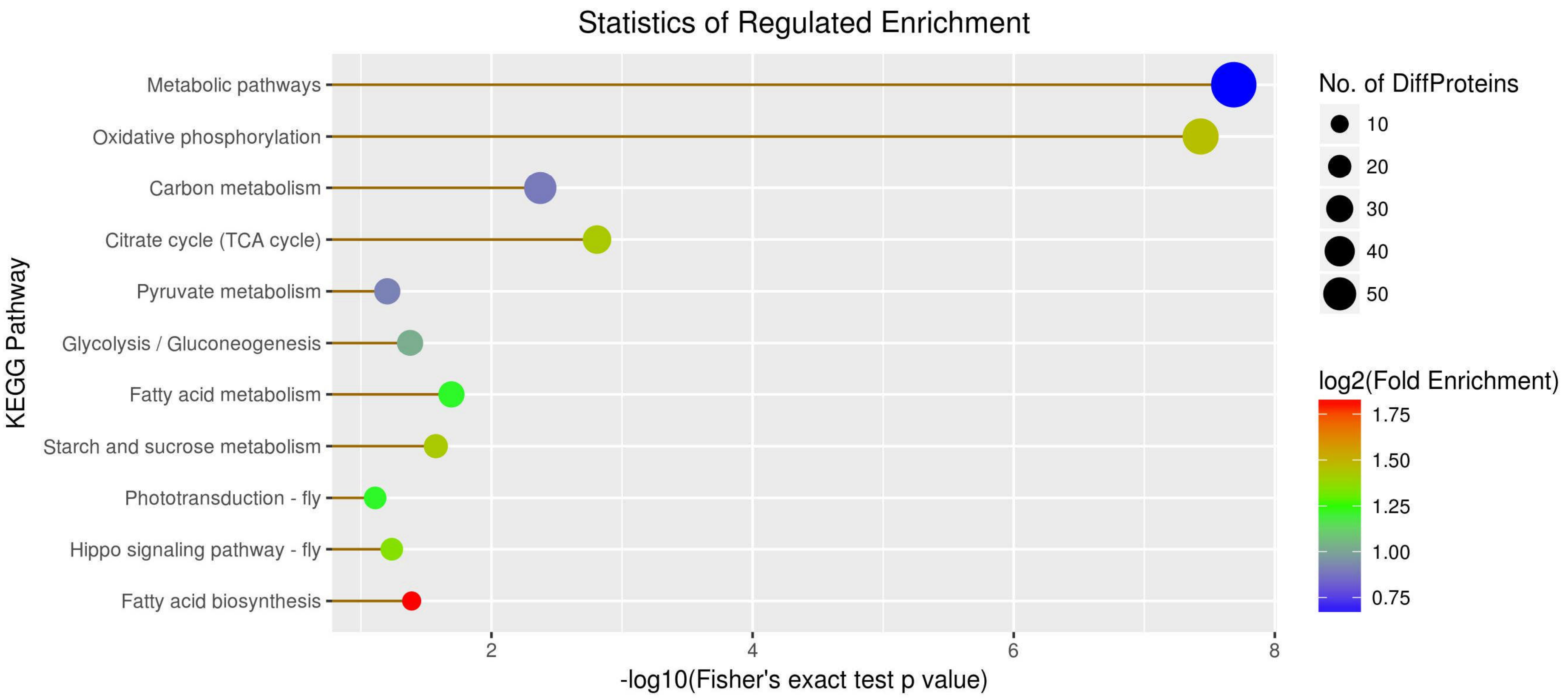

Supplementary figure 7. KEGG pathway enrichment analysis of differentially expressed proteins in AW vs. BW.

The pathway enrichment statistical analysis was performed by Fisher's exact test. When the p value is 0.05, it is considered that there is significant enrichment in this KEGG pathway.

Supplementary figure 8. Protein overview of complete metabolism in biological systems in AW vs. BW, map01100[75-77]

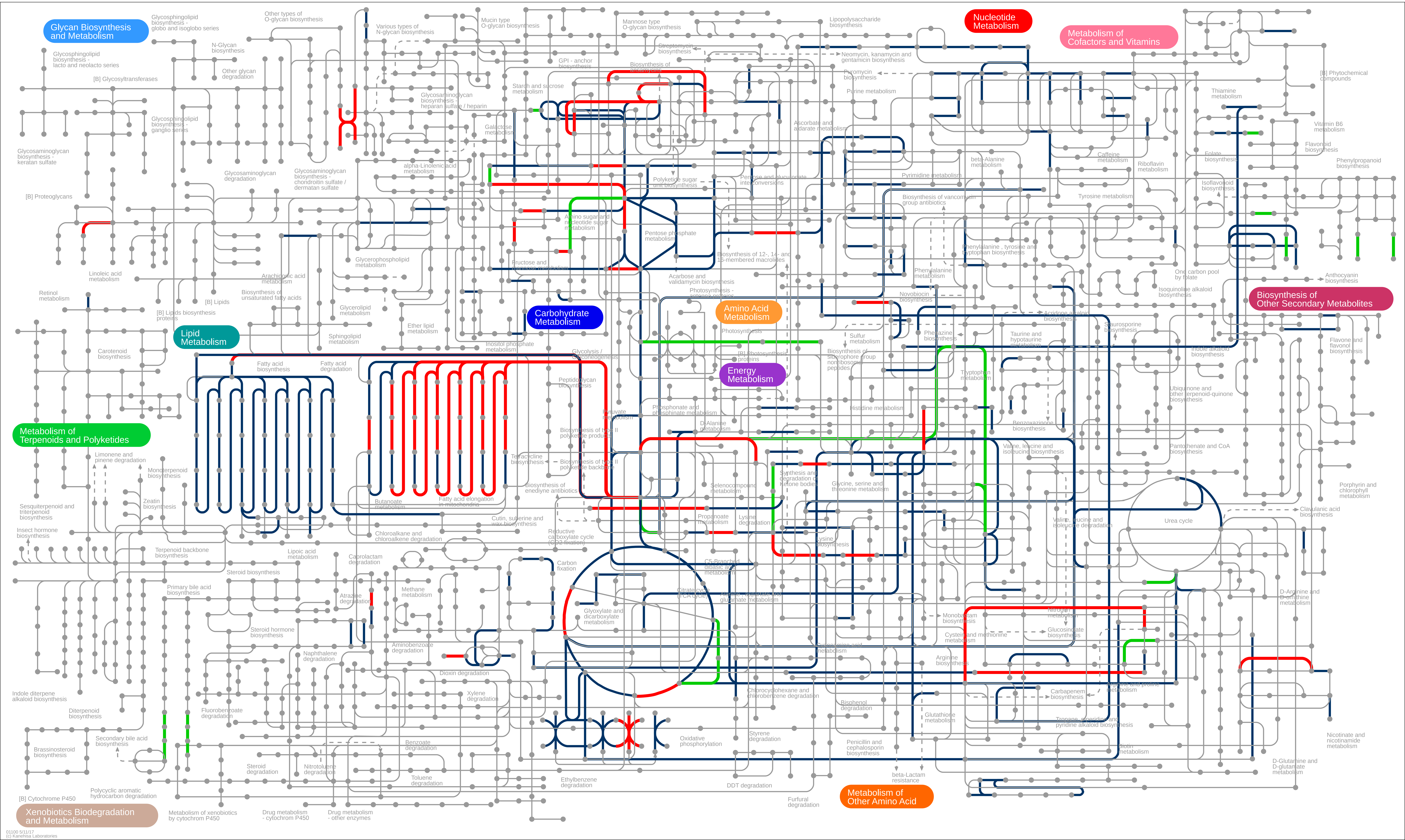

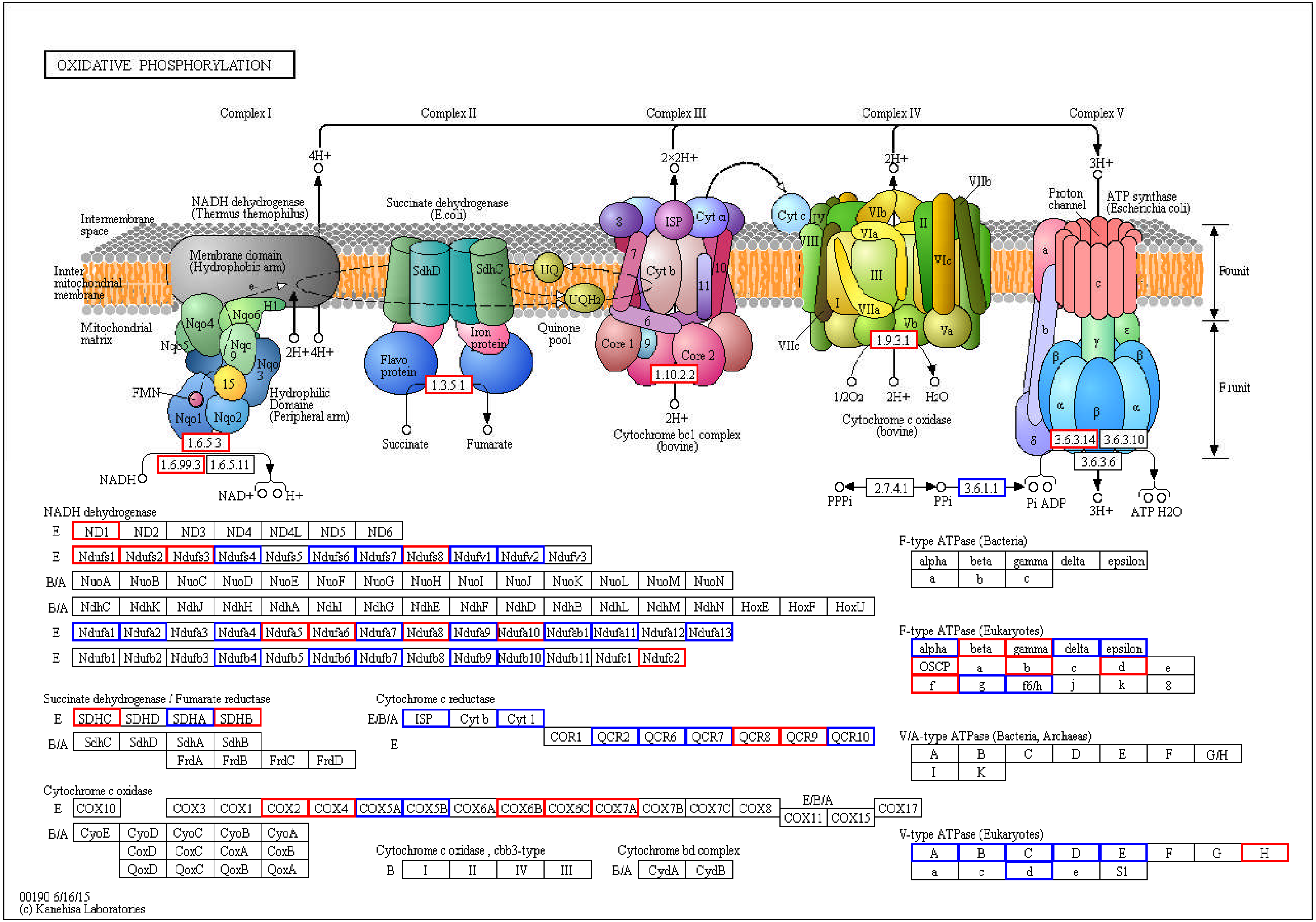

Supplementary figure 9. Annotated KEGG map for oxidative phosphorylation in AW vs. BW, map00190[75-77]

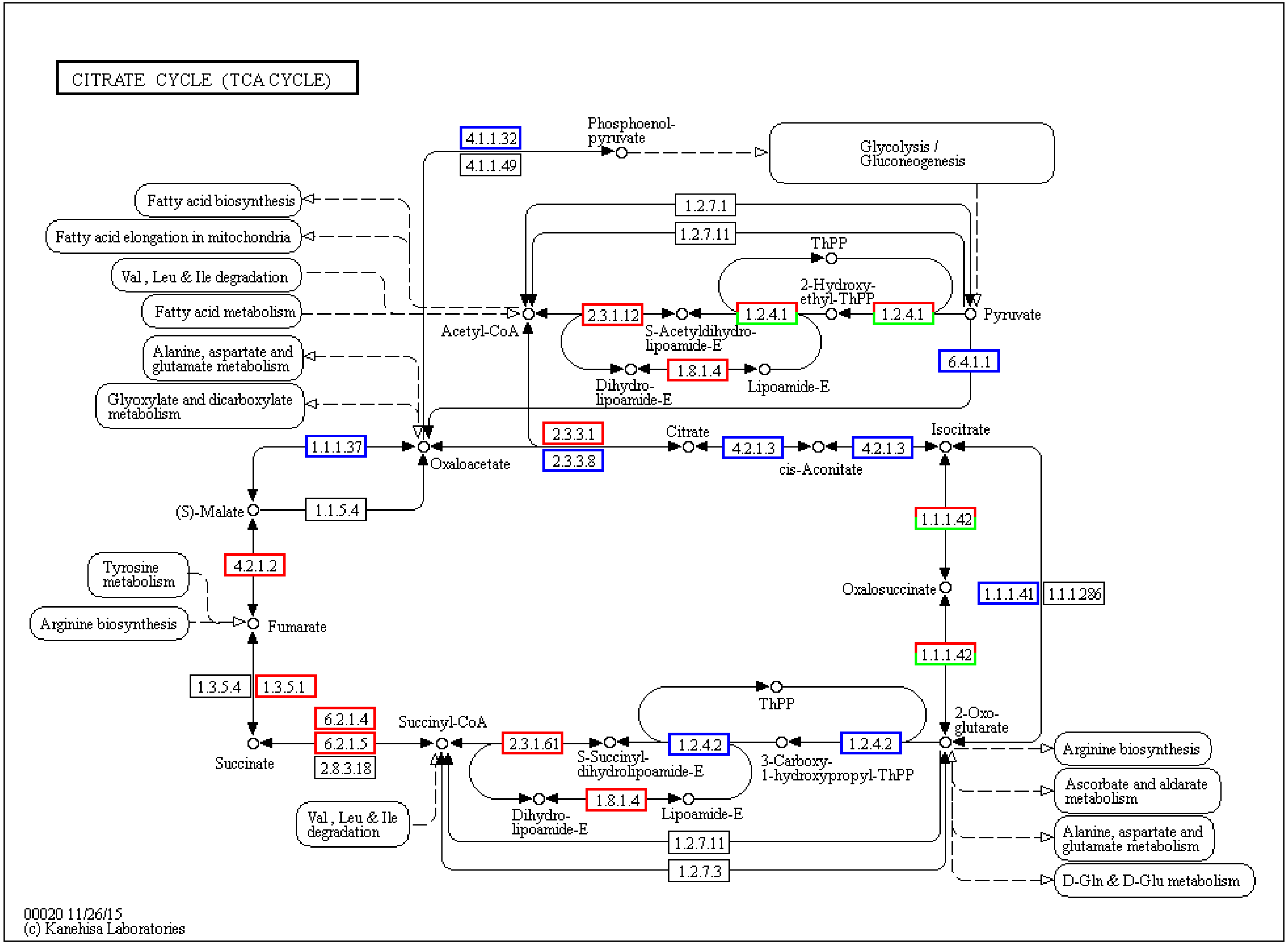

Supplementary figure 10. Annotated KEGG map for citrate cycle (TCA cycle) in AW vs. BW, map00020[75-77]

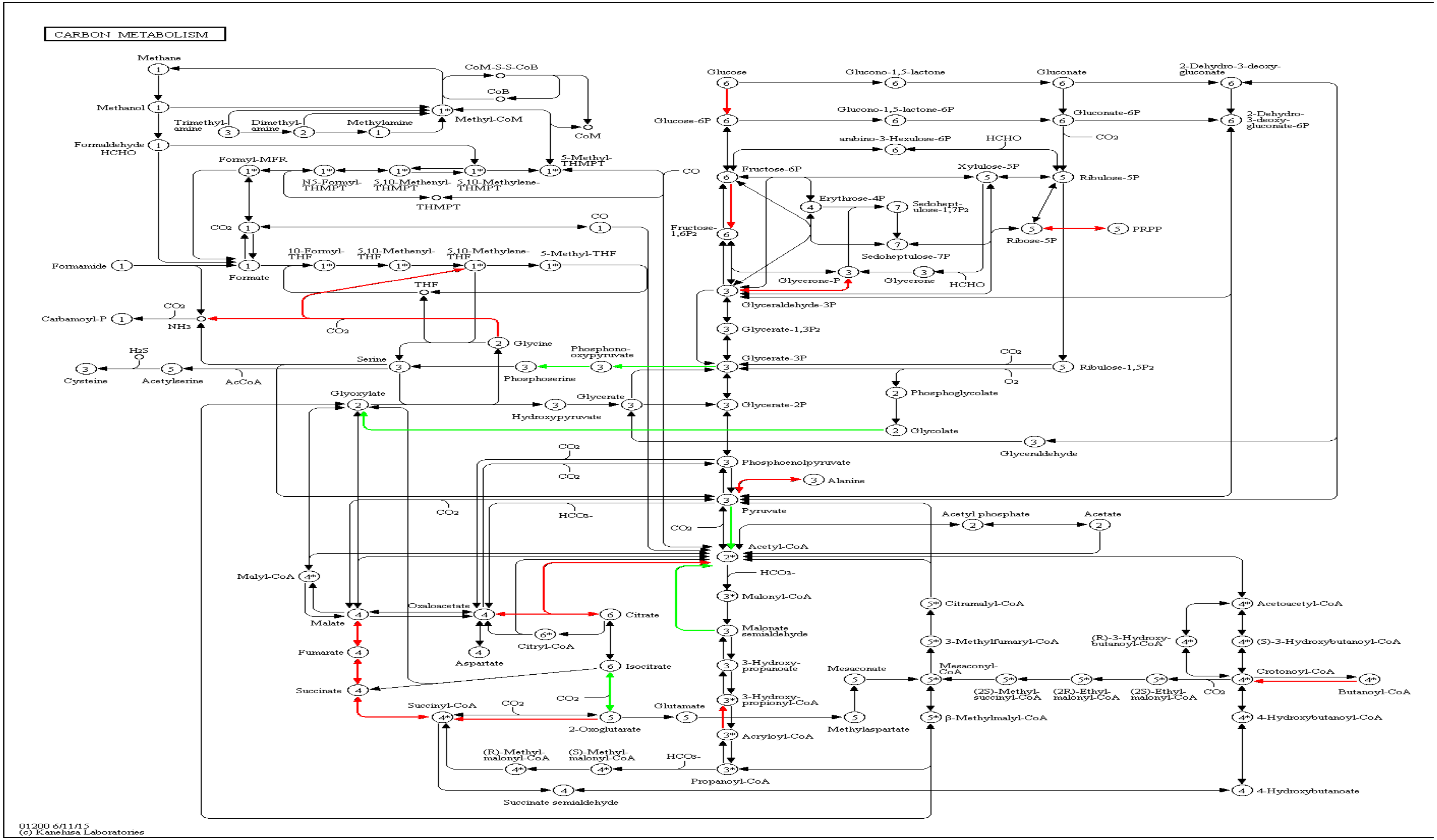

Supplementary figure 11. Annotated KEGG map for carbon metabolism in AW vs. BW, map01200[75-77]

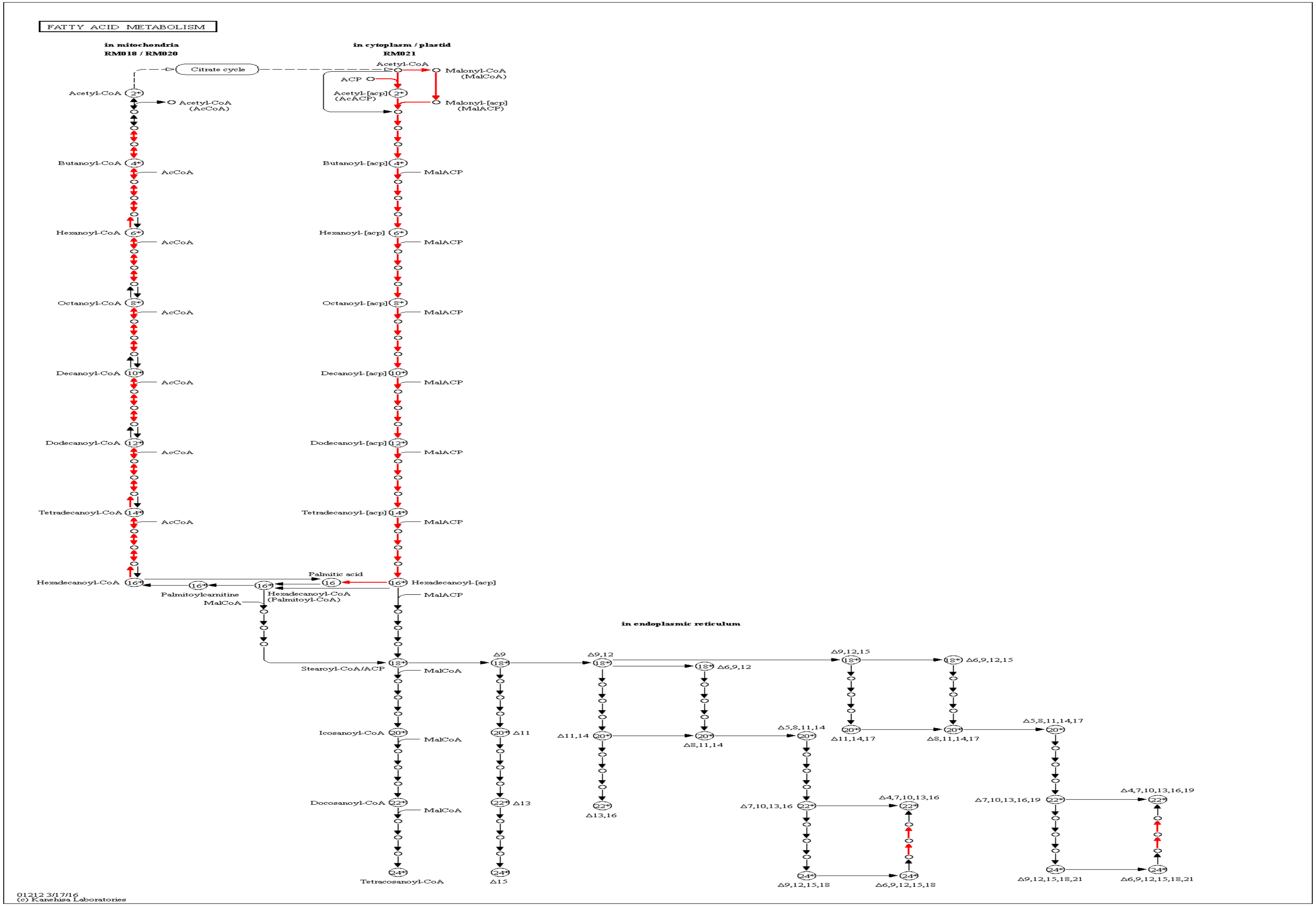

Supplementary figure 12. Annotated KEGG map for fatty acid metabolism in AW vs. BW, map01212[75-77]

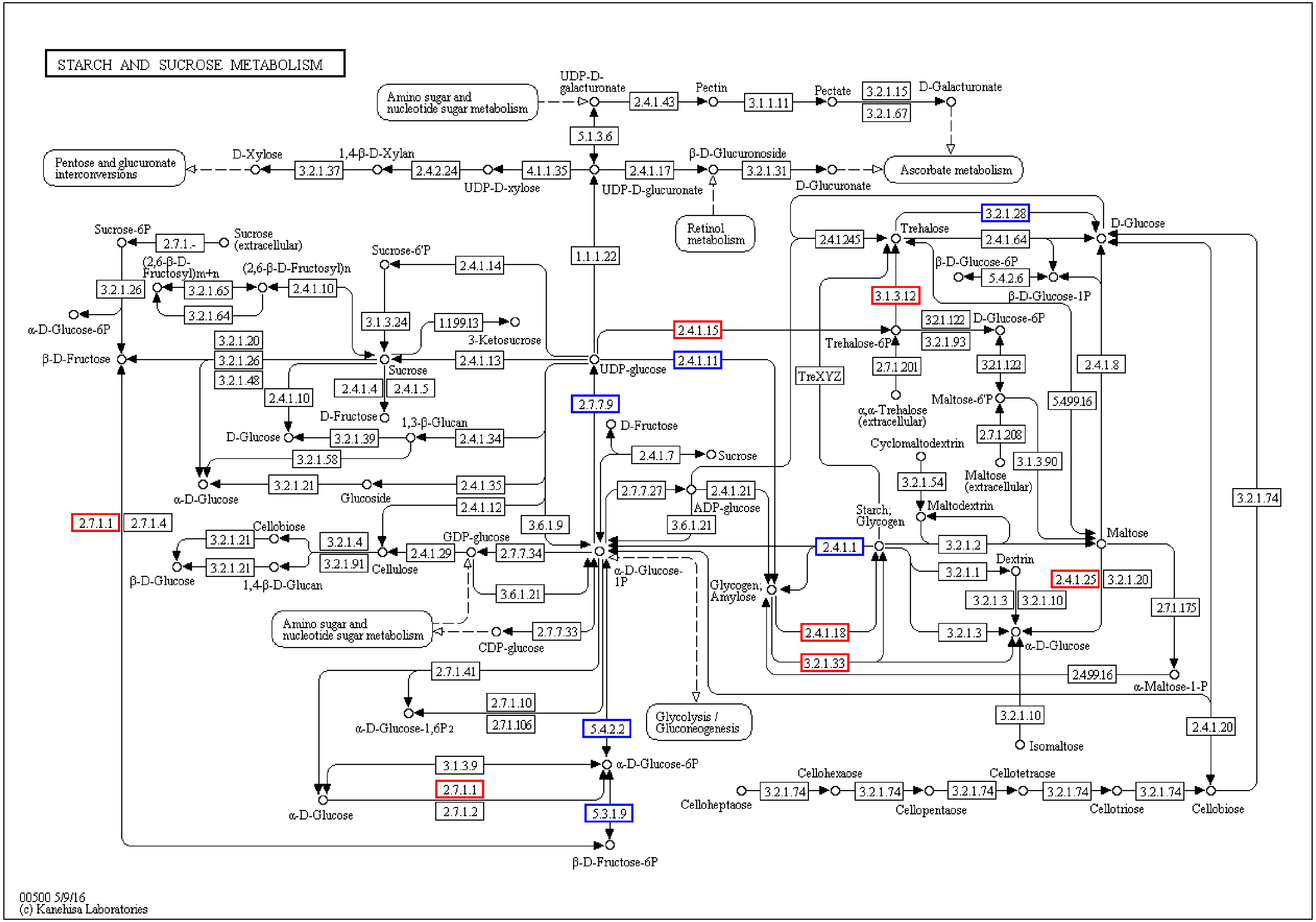

Supplementary figure 13. Annotated KEGG map for starch and sucrose metabolism in AW vs. BW, map00500[75-77]

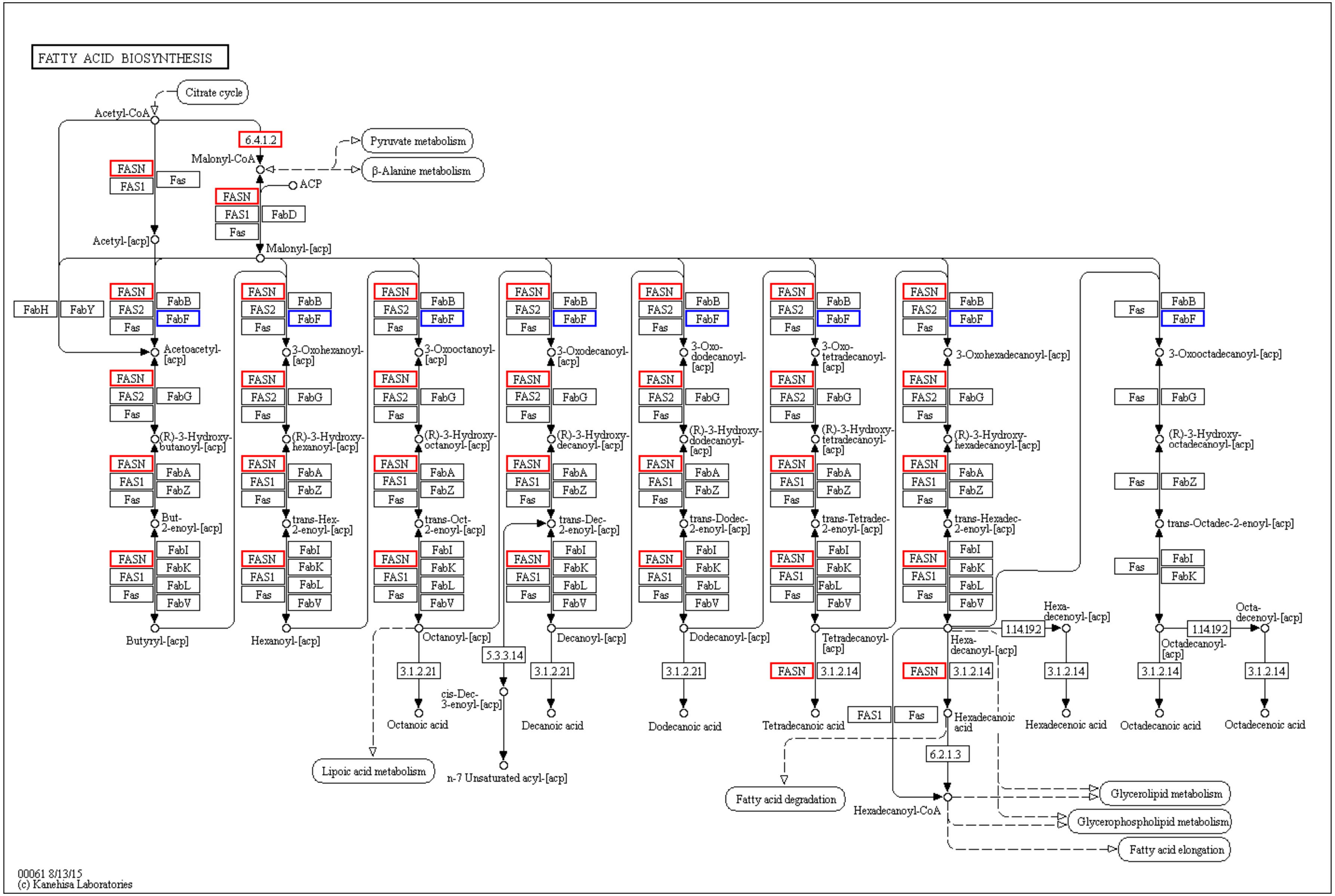

Supplementary figure 14. Annotated KEGG map for fatty acid biosynthesis in AW vs. BW, map00061[75-77]

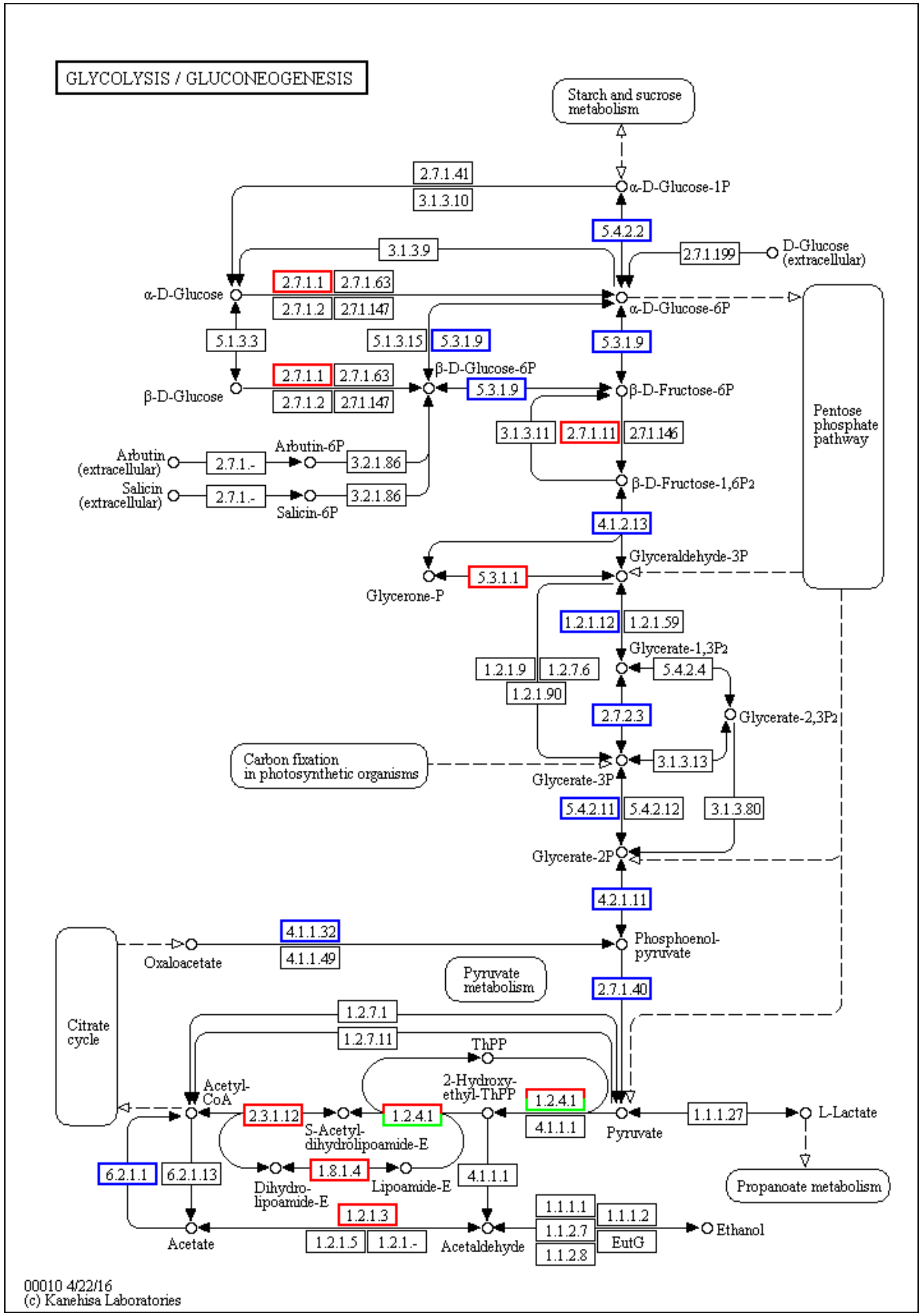

Supplementary figure 15. Annotated KEGG map for glycolysis/gluconeogenesis in AW vs. BW, map00010[75-77]

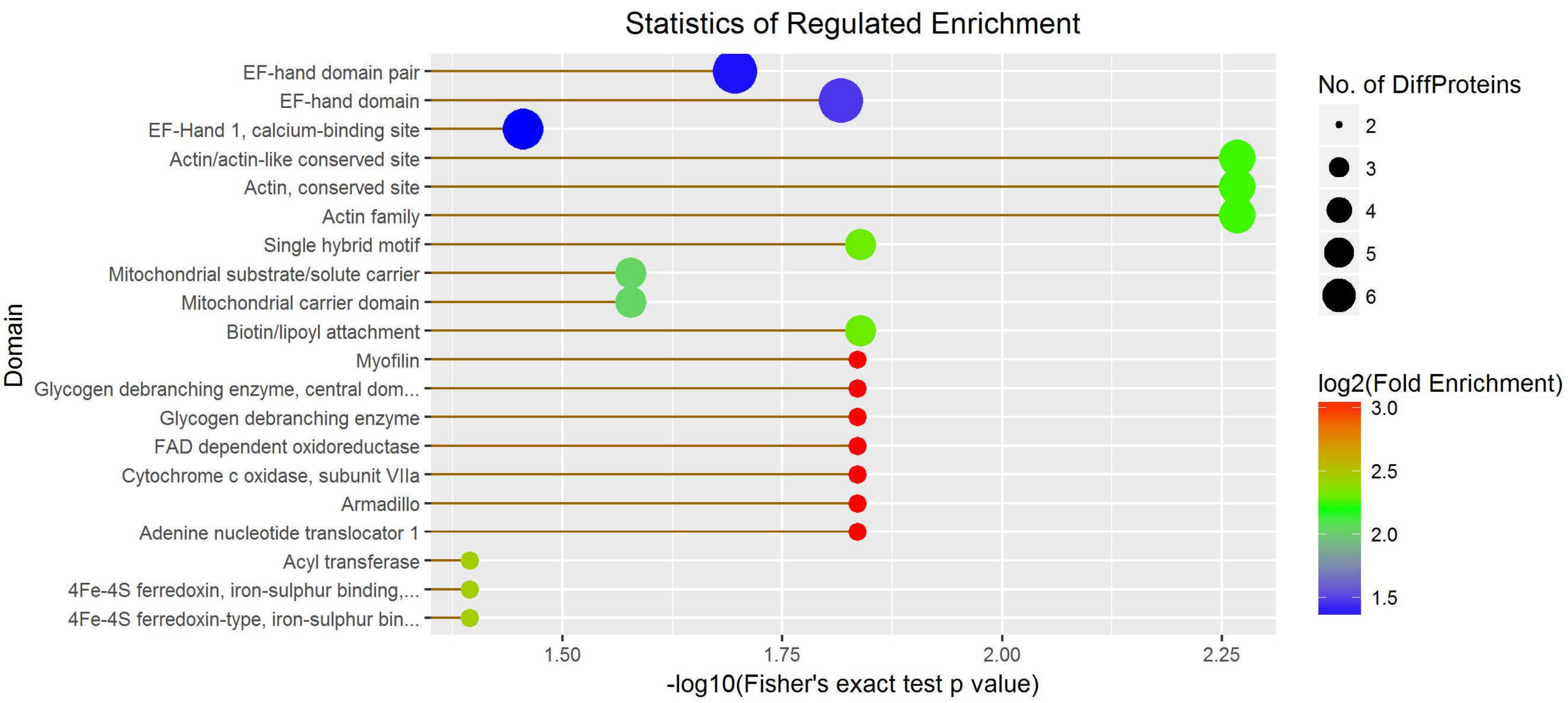

Supplementary figure 16. Visualization of significantly enriched Domain in AW vs. BW.

Upregulated proteins in functional categorizations of proteins differently expressed according to Fisher's extract test. The Number of Diffproteins is the number of differentially expressed proteins enriched in the Domain; the Domain enriched fold is shown on a log2 scale in a colour gradient.

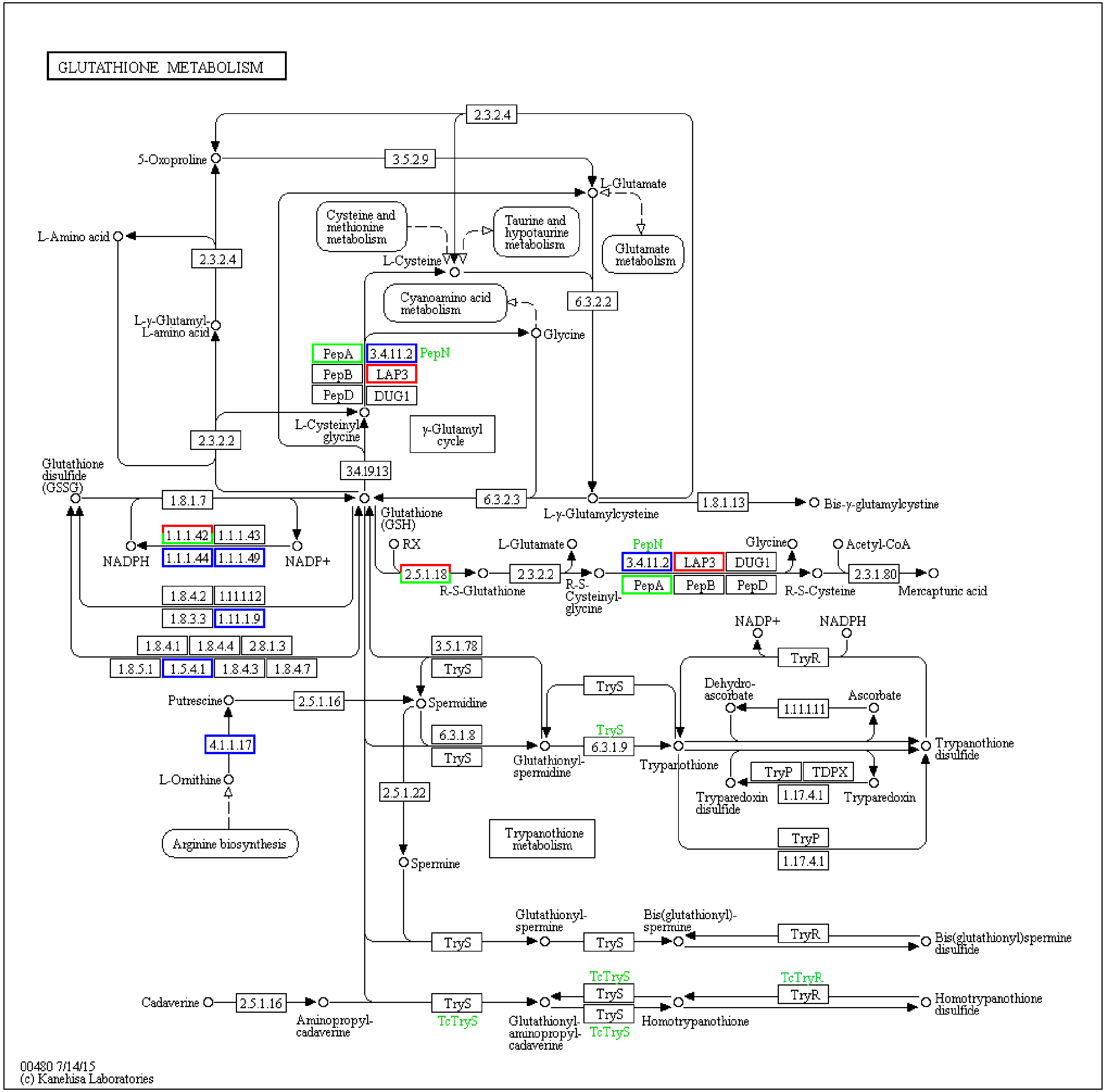

Supplementary figure 17. Annotated KEGG map for glutathione metabolism in AW vs. BW, map00480[75-77]
